# Physiological and genomic signatures of evolutionary thermal adaptation in redband trout from extreme climates

**DOI:** 10.1111/eva.12672

**Published:** 2018-07-20

**Authors:** Zhongqi Chen, Anthony P. Farrell, Amanda Matala, Nicholas Hoffman, Shawn R. Narum

**Affiliations:** ^1^ Hagerman Fish Culture Experiment Station Aquaculture Research Institute University of Idaho Hagerman Idaho USA; ^2^ Columbia River Inter‐Tribal Fish Commission Hagerman Idaho USA; ^3^ Department of Zoology The University of British Columbia Vancouver British Columbia Canada; ^4^ Faculty of Land and Food Systems The University of British Columbia Vancouver British Columbia Canada

**Keywords:** cardiac function, climate change, evolution, *Oncorhynchus mykiss*, population genomic, RAD‐seq, salmonid

## Abstract

Temperature is a master environmental factor that limits the geographical distribution of species, especially in ectotherms. To address challenges in biodiversity conservation under ongoing climate change, it is essential to characterize relevant functional limitations and adaptive genomic content at population and species levels. Here, we present evidence for adaptive divergence in cardiac function and genomic regions in redband trout (*Oncorhynchus mykiss gairdneri*) populations from desert and montane streams. Cardiac phenotypes of individual fish were measured in the field with a custom‐built electrocardiogram apparatus. Maximum heart rate and its rate limiting temperature during acute warming were significantly higher in fish that have evolved in the extreme of a desert climate compared to a montane climate. Association mapping with 526,301 single nucleotide polymorphisms (SNPs) across the genome revealed signatures of thermal selection both within and among ecotypes. Among desert and montane populations, 435 SNPs were identified as putative outliers under natural selection and 20 of these loci showed significant association with average summer water temperatures among populations. Phenotypes for cardiac performance were variable within each ecotype, and 207 genomic regions were strongly associated with either maximum heart rate or rate limiting temperatures among individuals. Annotation of significant loci provided candidate genes that underlie thermal adaptation, including pathways associated with cardiac function (IRX5, CASQ1, CAC1D, and TITIN), neuroendocrine system (GPR17 and NOS), and stress response (SERPH). By integrating comparative physiology and population genomics, results here advance our knowledge on evolutionary processes of thermal adaptation in aquatic ectotherms.

## 1 INTRODUCTION

Temperature is a master environmental factor that influences the distribution and diversity of species. Currently, the earth is experiencing rapid environmental changes and an associated global‐scale loss in biodiversity (Thomas et al., 2004). To respond to changing conditions, species need to utilize phenotypic plasticity to survive, shift their distribution through dispersal to more suitable habitats, or adapt based on standing genetic variation to avoid extirpation in unsuitable environments. To be able to predict ecological consequences of climate change, our understanding of thermal adaptation must be linked broadscale patterns to underlying mechanisms (Somero, 2012). For example, high‐level phenomena, such as changes in geographical distribution and life history, require a cause–effect explanation which is often answered by studying physiological limitations (Cooke et al., 2013). Furthermore, capability of adjusting physiological limits depends on underlying genetic variation, which is essential in predicting the adaptive potential and conducting genetic rescue (Urban et al., 2016). However, studies that integrate both physiology (Farrell, 2016; Pörtner, Bock, & Mark, 2017; Somero, 2011) and population genomics (Allendorf, Hohenlohe, & Luikart, 2010; Franks & Hoffmann, 2012; Narum, Buerkle, Davey, Miller, & Hohenlohe, 2013) to elucidate mechanisms of thermal adaptation in natural ecosystems remain scarce.

Physiological limitation in thermal performance and tolerance has been proposed to be related to cardiac function in a variety of fish species and especially salmonids (Casselman, Anttila, & Farrell, 2012; Eliason et al., 2011; Farrell, 2009; Pörtner et al., 2017). The heart is a life‐supporting organ that generates pressure to circulate blood, which transports oxygen (O_2_) from the gills of fishes to tissues where it removes metabolic wastes. Under warming conditions, O_2_ content of water decreases somewhat and yet organisms’ demand of O_2_ increases dramatically as a direct result of a thermodynamically derived increases in metabolic rate. Therefore, the circulatory system must work harder to meet the elevated O_2_ demand. However, the heart must function aerobically in the long‐term and has functional limitations, which likely sets the limit of O_2_ delivery capacity and whole organism thermal optima and maxima (Farrell, 2009). These ideas are embedded in the oxygen‐ and capacity‐limited thermal tolerance (OCLTT) hypothesis (Pörtner & Farrell, 2008; Pörtner et al., 2017). One empirical example of OCLTT is the local adaptation of sockeye salmon populations to migration temperatures (Eliason et al., 2011). However, traditional methods of characterizing OCLTT require measurements of both standard and maximum metabolic rate, which are time‐consuming and therefore not ideal for field surveys of most natural populations. Casselman et al. (2012) showed that thermal response of maximum heart rate (*f*_h,max_) has some critical rate transition indices (e.g., Arrhenius breakpoint temperature (*T*_AB_) and temperature of peak *f*_h,max_ (*T*_PEAK_)) and can be used as a useful surrogate for classic metabolic rate approaches (Casselman et al., 2012). Measurement of *f*_h,max_ can be taken in a more timely fashion with simple equipment and therefore can be used as a relatively fast screening tool in field settings (Chen et al., 2015; Drost, Carmack, & Farrell, 2014). Because of the vital role of cardiac function in thermal tolerance and performance, identifying the genetic architecture of cardiac thermal performance and tolerance could help elucidate evolutionary processes of thermal adaptation.

Thermal performance and tolerance‐related traits are heritable in salmonids (Muñoz, Farrell, Heath, & Neff, 2015) and controlled by many genes (Jackson et al., 1998; Quinn, McGowan, Cooper, Koop, & Davidson, 2011). Variation in functionally important genes underlying adaptive traits could be translated into differences in performance and life‐time fitness, which further affect the frequency of these genes in following generations (Franks & Hoffmann, 2012). In differing environments, selection may alter the frequency of beneficial gene variants causing signals of adaptive divergence among populations. Many methods have been developed to detect loci and patterns of divergent selection including outlier tests and genome–environmental association tests that account for neutral genetic structure in natural populations (Beaumont & Balding, 2004; Duforet‐Frebourg, Luu, Laval, Bazin, & Blum, 2016; Foll & Gaggiotti, 2008; Hoban et al., 2016; Whitlock & Lotterhos, 2015). Additionally, association mapping of genomic regions can provide strong evidence for the genetic basis of specific traits (Everett & Seeb, 2014; Jackson et al., 1998). In some cases, high marker density may be necessary to resolve candidate genomic regions with these approaches; therefore, advances in molecular technology provide opportunities to capture a large number of genomic markers to help understand mechanisms of adaptation (Baird et al., 2008; Narum, Buerkle et al., 2013).

Redband trout (*Oncorhynchus mykiss gairdneri*) in the interior Columbia River of the United States occupy streams with diverse environments that differ greatly in temperature (Narum, Campbell, Kozfkay, & Meyer, 2010; Rodnick et al., 2004). Summer water temperature in some desert streams can reach 29°C, which is close to the upper thermal tolerance limits of *Oncorhynchus* and definitely exceeds their thermal optima, whereas the water temperature in some montane streams rarely exceeds 20°C (Gamperl et al., 2002; Rodnick et al., 2004; Zoellick, 1999). Thermal performance has been examined from animals acclimated in both laboratory (Chen, Farrell, Matala, & Narum, 2018; Narum, Campbell, Meyer, Miller, & Hardy, 2013) and field environments (Gamperl et al., 2002; Rodnick et al., 2004), but with limitations in the number of populations and physiological measurements. In this study, we sampled six populations of redband trout across diverse environmental conditions that ranged from hot desert, through cool montane to cold montane streams and measured cardiac function of individual fish during acute warming with a custom‐built electrocardiogram (ECG) apparatus in the field. Results show that *f*_h,max_ and its rate transition temperatures during warming were correlated with habitat climate and cardiac performance was the best in populations from warm environments. We also identified candidate genomic regions for thermal adaptation among populations and those that were associated with phenotypic variation in *f*_h,max_ within ecotypes. Overall, this study contributes valuable knowledge to the mechanistic basis of evolutionary thermal adaptation in aquatic ectotherms by integrating comparative physiology and population genomic approaches.

## 2 MATERIALS AND METHODS

### 2.1 Study populations

**Figure 1:**
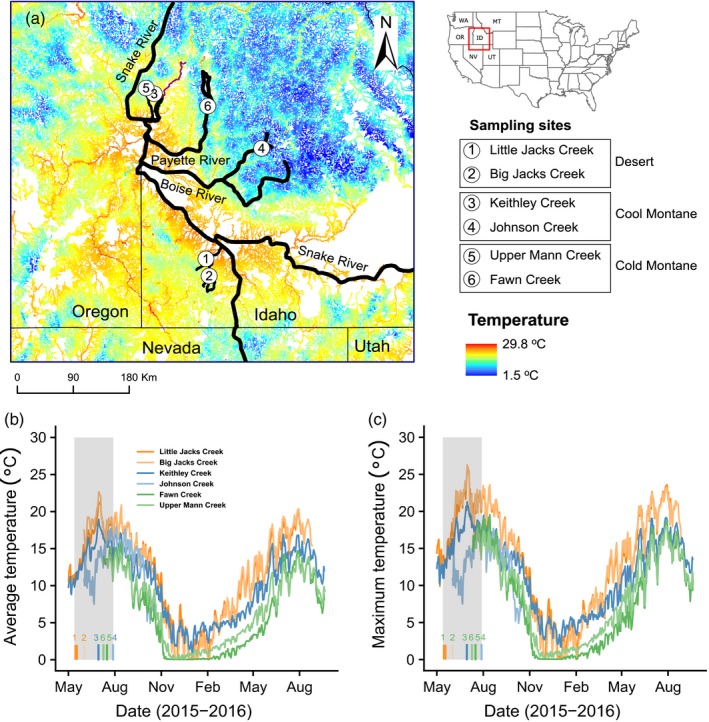
Location and thermal regime of sampling sites. (a) geographical locations of six sampling sites and modeled mean stream temperature in August (Isaak et al., 2016); (b) average stream temperature (daily); (c) maximum stream temperature (daily). Note that data of Johnson Creek in panel b and c were from 2009. Shaded region in gray represents the range of sampling dates for cardiac phenotypes and genetic tissues, with specific sampling time for each location noted by a colored block

Interior Columbia River redband trout can be classified into three ecotypes according to their geographical characteristics and thermal regimes: desert, cool montane, and cold montane (Meyer, Lamansky, & Schill, 2010; Narum et al., 2010). Desert ecotypes are found in arid desert regions, where summer air temperature can reach over 40°C and water temperature over 25°C. Cold montane ecotypes are adapted to high elevation and forested montane streams, where summer water temperature rarely exceeds 20°C. Cold Montane streams also have long winter duration with water temperature close to freezing point. Redband trout adapted to montane streams where thermal regime is between desert and cold montane are classified as cool montane ecotype. Phylogeny of populations in this study has also been examined previously (Narum et al., 2010) to help classify populations into ecotypes genetically. Redband trout from this study were sampled from six streams in four tributaries of Snake River in southern Idaho, USA (Figure 1). Desert ecotype redband trout were sampled from Big Jacks Creek (42.561775; ‐116.039680) and Little Jacks Creek (42.750433, −116.091990. Cool montane fish were sampled from Keithley Creek (44.51787, −116.83524) and Johnson Creek (43.939104, −115.283277). Cold montane fish were sampled from Upper Mann Creek (44.57841, −116.95333) and Fawn Creek (44.38714, −116.0615). One‐year‐old fish were sampled using a backpack electroshocker (LR‐20B, Smith‐Root, Vancouver, WA, USA) with age estimated based on size classes for each stream. Sampling efforts occurred at each location with stream temperatures ranging from 11.6 to 18.8°C at time of fish capture, and then fish were held in containers with cold aerated water (<12°C) for a minimum of one hour but no more than ten hours prior to experiments. Sampling fish from natural creeks for this study was reviewed and approved by Idaho Department of Fish and Game (Permit #F‐13‐06‐15) following approved animal care and use protocol (University of Idaho IACUC 2013‐80). Water temperature for each stream was obtained using 2–3 temperature loggers (TidbiT v2, onset®, Bourne, MA, USA) with recording intervals of 25–30 min. Water temperature data were not available for Johnson Creek because of a forest fire in 2016 that prevented access to recover data loggers, but previous data from 2009 were available.

### 2.2 Field measurement of cardiac phenotypes

**Figure 2:**
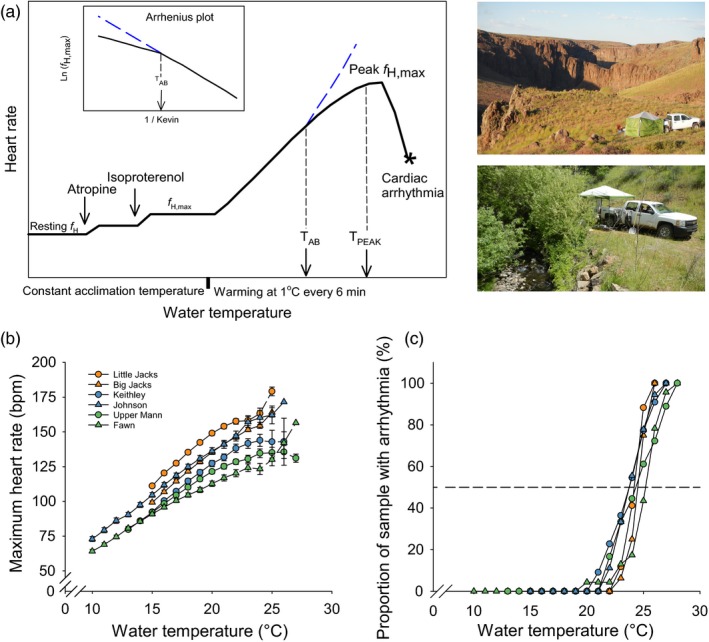
Intraspecific maximum heart rate (*f*_h,max_) during acute warming in six populations of redband trout. (a) Diagram to demonstrate how the cardiac phenotypes were methodologically determined and photographs of the field setup of the electrocardiogram apparatus for cardiac phenotyping. (b) thermal response of *f*_h,max_, which were correct to a common mass of 11 g using mass exponent of −0.1. (c) cumulative proportion of samples reaching peak *f*_h,max_

A custom‐built ECG apparatus was designed to examine cardiac phenotypes in field conditions following protocols from previous studies (Casselman et al., 2012; Chen et al., 2018). Briefly, each fish was anesthetized using 80 mg/L MS222 buffered with 160 mg/L NaHCO_3_ (Sigma‐Aldrich, St. Louis, MO, USA). A small piece of fin tissue was sampled before fish was placed (submerged in water) in the ECG measuring system. Anesthetized fish were stabilized at the initial test temperature for 0.5 hr. After stabilization for one hour, fish were intraperitoneal injected with atropine sulfate (2.7 mg/kg, Sigma‐Aldrich, St. Louis, MO, USA) and isoproterenol (9 μg/kg, Sigma‐Aldrich, St. Louis, MO, USA). Each injection was given 15 min to take effect and together resulted in an elevated and stabilized *f*_h,max_ (Figure 2a). Then, water temperature was increased at the rate of 1°C every six min. Warming was continued until peak *f*_h,max_ is reached, which is when either *f*_h,max_ starts decreasing dramatically (typically >5 bpm over 1°C warming, Figure 2a) or the heartbeat developed an arrhythmia (usually a missed ventricular depolarization, which can be visualized as a missed QRS complex in the ECG). The temperature of peak *f*_h,max_ was recorded as *T*_PEAK_. Once *T*_PEAK_ was reached, each fish was immediately removed from the chamber and recovered before it was released back to the creek. In an Arrhenius plot of log‐transformed *f*_h,max_ (ln(*f*_h,max_)) against inverse Kelvin temperature (1/K), the first *T*_AB_ was estimated from the intersection of two best fit linear regression lines (Yeager & Ultsch, 1989) (Figure 2a). Difference in *T*_AB_ among populations was compared by Kruskal–Wallis one‐way ANOVA on Ranks with Dunn's *post hoc* tests. Differences in *f*_h,max_ at 20°C, Peak *f*_h,max_ and temperature coefficient (Q_10_) among populations were compared by One‐way ANOVA with Holm–Sidak *post hoc* tests. Significance was considered at *p* < 0.05.

### 2.3 Sequencing and genotyping

DNA was extracted from dried fin tissues using a DNeasy Blood and Tissue Kit (QIAGEN, Valencia, CA). Restriction‐site‐associated DNA (RAD) Sequencing libraries were prepared using methods previously outlined (Baird et al., 2008; Miller et al., 2012) with restriction enzyme *Pst‐*I‐HF (NEB, Ipswich, MA, USA). A total of 115 individually barcoded individuals were sequenced. Sequencing strategy was designed with 5–10 barcoded individuals per library to yield a minimum of 20 million reads per fish to achieve average locus read depth >20 (i.e., estimated 732,000 *Pst*‐I cut sites in a 3.0 Gb genome). Libraries were sequenced on an Illumina HiSeq 1500 for 100 cycles across a total of 45 lanes of SR100 flow cells to achieve minimum target of 20 million reads per individual.

Single nucleotide polymorphism (SNP) loci were genotyped *de novo* by constructing a SNP catalog (see Chen et al. (2018) for details). This was performed using the software pipeline Stacks (Catchen, Hohenlohe, Bassham, Amores, & Cresko, 2013). Sequence reads were truncated to 75 bases by removing the 3′ sequence that was most prone to error. After individual sample reads were quality filtered and demultiplexed, sequences for each sample were used for SNP discovery. A *de novo* catalog of RAD tag SNPs was created by selecting two individuals from each population with at least three million (M) reads to evenly represent genetic variation throughout the source populations. Individual samples were then aligned to the catalog and genotyped. Genotypes were filtered to exclude (a) any RAD tag locus with more than four SNP sites to remove putative paralogous sequence variants, hypervariable, or poorly sequenced tags, (b) any SNP marker with more than two alleles to remove SNPs with sequencing errors or loci that do not fit a biallelic statistical model, (c) any SNP marker missing more than 25% of the genotypes across all of the populations to limit the amount of missing data, and (d) any SNP marker with an average minor allele frequency falling below 0.02 in any of the populations to exclude spurious rare SNPs or sequencing errors.

### 2.4 Outlier loci and population structure

We used three tools that considered neutral structure while trying to be conservative throughout the analyses. To this end, OutFLANK V0.1 estimated the distribution of neutral loci by trimming extreme *F*_ST_ values (Whitlock & Lotterhos, 2015). We ran the program with default options: minimum heterozygosity of 0.1, left and right trim fraction of 0.05. In addition, an *F*_ST_ outlier approach was based on a hierarchical Bayesian model (Beaumont & Balding, 2004) with a reversible jump Markov chain Monte Carlo, implemented in BayeScan V2.1 software (Foll & Gaggiotti, 2008). We did the analyses on 20 pilot runs with 5,000 iterations, followed by 50,000 iterations with a burn‐in length of 50,000 iterations. Outlier loci were considered candidates for selection if they had a minimum Bayes factor of 3 and false discovery rate (FDR) of 0.1. Lastly, PCAdapt V3.0.3 (Duforet‐Frebourg et al., 2016) detected candidate outliers using principal component analyses (PCA) to account for population structure. PCAdapt examines the correlation between SNPs and the first *k* principal components by running a communality test to identify SNPs that are significantly associated with the population differentiation as outliers. The *k* value was set to 3 according to the scree plot. After performing outlier tests, test significance values from OutFLANK and PCAdapt were examined using histograms, calibrated according to their genomic inflation factor (François, Martins, Caye, & Schoville, 2016). FDR was corrected by Benjamini–Yekutieli FDR (Benjamini & Yekutieli, 2001).

PCA was performed using the algorithm in R package “adegenet” (Jombart, 2008) on a matrix of allele frequencies that was preprocessed by replacing the missing data with mean allele frequency. Heterozygosity was calculated using GENEPOP for each locus (Raymond & Rousset, 1995) and then mean value across loci. Patterns of isolation by fluvial distance and isolation by summer stream temperature were analyzed using Mantel test in GENEPOP (Raymond & Rousset, 1995).

### 2.5 *F*_ST_ within ecotypes for each locus

In the analysis for each phenotype, individuals from each population were ranked for each phenotype (*T*_AB_, *f*_h,max_ at 20°C, peak *f*_h,max_, and *T*_PEAK_) and evenly divide into two phenotypic groups (low and high). Only loci with genotyping success rate of 75% within each ecotype were kept for *F*_ST_ analysis. The *F*_ST_ between phenotypic classes were calculated for sliding windows across the entire genome (window size of 10 kb, step size of 200 bp) using an approach called local score that accounts for physical linkage (Fariello et al., 2017). Significance level of the *F*_ST_ for sliding windows was determined by first calculating the *p*‐values for each marker using Fisher's exact test implemented in R statistical packages (R Core Team, 2017), and then, *p*‐values were combined for each window (window size of 10 kb, step size of 200 bp) using weighted *z*‐score transformation method (Whitlock, 2005) implemented in R Package “survcomp” (Schröder, Culhane, Quackenbush, & Haibe‐Kains, 2011). Significant threshold for sliding window *F*_ST_ was determined by Benjamini–Yekutieli FDR (Benjamini & Yekutieli, 2001).

### 2.6 RAD tag and gene annotation

RAD tag sequences were aligned to the rainbow trout genome (Omyk_1.0; GenBank accession: GCA_002163495.1, USDA/ARS) (S. Lien and Y. Palti, unpublished, 2017) using program Bowtie2 v.2.2.4 (Langmead & Salzberg, 2012) to obtain chromosomes assignment and genomic positions. By querying the rainbow trout genome for coding sequence within 5 kb of alignment SNP sites, putatively linked genes were identified. For the sliding window, *F*_ST_, only genes within the sliding window were retrieved. To identify gene functions and annotations, coding sequences were then queried against the NCBI nucleotide sequence database.

## 3 RESULTS

### 3.1 Differences in habitat thermal regimes

Native redband trout in this study represent ecologically divergent populations from environments classified as desert, cool montane, and cold montane. Desert streams had the highest mean and maximum water temperatures (Figure 1b,c). In the summer of 2015, water temperature in Little Jacks Creek and Big Jacks Creek reached 26°C, while Fawn Creek and Upper Mann Creek never reached 20°C. Little Jacks Creek, Big Jacks Creek, and Fawn Creek had the greatest diurnal temperature fluctuations of over 7.5–7.8°C. The mean summer temperature of desert streams was 2.3°C higher than cool montane streams and by 5.1°C higher than cold montane streams.

### 3.2 Intraspecific variation in *f*_h,max_

With full adrenergic stimulation and vagus blockage, thermal sensitivity of maximum heart rate (*f*_h,max_) in response to acute warming was measured to characterize the adaptation of cardiac function (Figure 2a). Overall, fish increased *f*_h,max_ by 55% from an average of 97.7±0.9 bpm at 15°C (*N* = 114) to 142.8 bpm at 23°C (*N* = 88) (an average of 5.6 bpm for every degree Celsius warming, Figure 2b) accounting for the effect of body size on heart rate using a mass exponent of ‐0.1 (Chen et al., 2018). The desert ecotype, especially Little Jacks, had the highest *f*_h,max_, compared with the cool and cold montane ecotypes. During warming, the population‐specific differences in *f*_h,max_ among populations were maintained. Exceptions were noted between Keithley, Upper Mann, and Fawn populations that had the same *f*_h,max_ at 15°C, but the difference between them became larger as temperature increased (Figure 2b) because of the significantly different *Q*_10_ (Table 1). Another noticeable finding was the low phenotypic variation in *f*_h,max_ within populations, but larger variation among populations and ecotypes (Figure 2b).

**Table 1:** Summer thermal regimes and cardiac phenotypes of six redband trout populations

Pop^a^	Body size			Cardiac phenotypes^b^				
	N	Weight (g)	Length (mm)	*f*_h,max_ at 20°C^c^ (bpm)	Peak *f*_h,max_ (bpm)	*T*_AB_ d (^o^C)	*T*_PEAK_ e (^o^C)	*Q*_10_ f
LJ	17	14.4±1.1	114.0±3.6	149.0±1.6^A^	161.4±2.6^A^	20.7±0.2^A^ (16)	23.0±0.3	1.63±0.02^B^
BJ	16	11.7±0.8	103.7±2.5	135.4±1.6^B^	154.2±2.3^A^	20.6±0.3^A^ (13)	23.2±0.3	1.68±0.03^AB^
KC	22	10.0±0.6	100.1±2.2	127.3±2.2^C^	143.6±3.0^AB^	20.2±0.4^A^ (16)	22.5±0.4	1.79±0.04^A^
JC	18	14.9±1.4	113.7±3.7	136.3±2.4^B^	152.6±4.3^A^	18.3±0.3^BC^ (14)	22.9±0.4	1.61±0.03^B^
UM	18	4.6±0.4	76.7±1.7	121.5±1.6^C^	132.2±2.4^BC^	19.6±0.2^AB^ (16)	22.2±0.5	1.63±0.03^B^
FC	23	3.3±0.2	69.1±1.5	112.8±2.0^D^	125.4±3.4^C^	17.7±0.4^C^ (22)	23.9±0.4	1.46±0.03^C^

*Notes*

a Redband trout populations, LJ: Little Jacks; BJ: Big Jacks; KC: Keithley; JC: Johnson; UM: Upper Mann; FC: Fawn.

b All values are present as mean±s.e.m. Values with different superscripted uppercase letters are significantly different (*p* < 0.05).

c *f*_h,max_ at 20°C: maximum heart rate at 20°C.

d *T*_AB_: Arrhenius Breakpoint temperature. Numbers in parentheses are sample size for *T*_AB_ in each population.

e *T*_PEAK_: Temperature for peak *f*_h,max_.

f *Q*_10_: temperature coefficient for *f*_h,max_ during acute warming from 15°C to *T*_PEAK_.

Peak *f*_h,max_ significantly differed among populations (*p* < 0.05) and was positively related to environmental thermal regimes (Table 1). Furthermore, individual fish with a high *f*_h,max_ were also likely to reach a higher peak *f*_h,max_ (Supporting information Figure S1). However, this pattern was stronger in cool montane (*R* ^2^ = 0.44; *p* < 0.001) and cold montane fish (*R* ^2^ = 0.58; *p* < 0.001) than in desert fish (*R* ^2^ = 0.25; *p* = 0.003) which already had a higher peak *f*_h,max_.

Significant differences were also found in rate transition temperatures for *f*_h,max_ among ecotypes as estimated by Arrhenius breakpoint temperature for *f*_h,max_ (*T*_AB_) (Table 1). Similar to the aforementioned *f*_h,max_ results, Little Jacks trout had the highest *T*_AB_, while Fawn trout had the lowest *T*_AB_ (*p* < 0.05). This result suggests a positive association between *f*_h,max_ and *T*_AB_, which held true beyond the population level through to the individual level (Supporting information Figure S1). Although *T*_AB_ was higher than the average stream temperature in all populations (by 2.9–7.4°C), it was lower than the maximum stream temperature in all desert populations (by 2.7–3.0°C) and one cold montane population (Fawn) (by 1.9°C). In contrast, temperature of peak *f*_h,max_ (*T*_PEAK_) was not significantly different among populations and individual fish reached peak *f*_h,max_ over a wide temperature range of 20–28°C (Figure 2c; Table 1). *T*_PEAK_ was above the average stream temperature in all montane populations, but lower than the maximum temperature in desert populations, suggesting potential functional limitations in desert populations during summer months. There was no significant association between *T*_AB_ and *T*_PEAK_ within any ecotype (*R* ^2^ < 0.12; *p* > 0.05).

### 3.3 Genome scan for adaptive loci among populations

**Figure 3:**
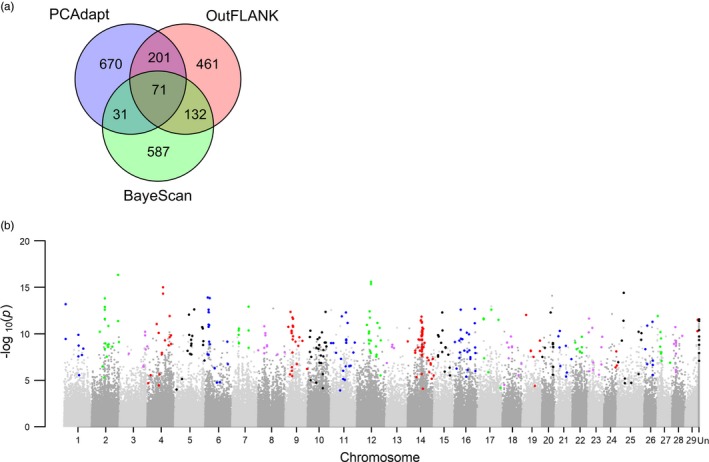
Outlier loci analyses. (a) number of outlier loci identified by each method. (b) Manhattan plot of combined *p*‐values, which were derived from the median *z*‐score of results from OutFLANK and PCAdapt. Only the loci that were identified by at least two methods are noted as outliers (a total of 435). Outlier loci are marked as nongray points in the Manhattan plot. Loci that were not assigned a chromosome position are marked as “Un.” Mapping details and annotation of outlier loci can be found in Supporting information Table S1

Using *Pst*‐I RAD sequencing, a total of 1,991,228 RAD tags were sequenced. After quality filtering, 526,301 biallelic SNP markers from 376,271 tags (75‐bp) were used for population genomic analyses. A dense coverage of SNP markers enabled us to identify genomic signatures underlying thermal selection. Of the three methods that we used to test for candidate outlier loci, PCAdapt identified 973 loci (0.19% of all marker) as outliers, BayeScan identified 821 loci (0.16%) and OutFLANK identified 865 (0.16%) (Figure 3a). To reduce the number of false positives, only those loci identified by at least two tools were considered as outliers, which totaled 435 SNPs (0.083%) from 389 RAD tags (Figure 3b). Although outliers were found in all chromosomes, they were most abundant in chromosome Omy14 (12.2%). Heterozygote deficiency was observed in all populations and is stronger in outlier loci than neutral loci. The expected heterozygosity for outliers was higher in desert populations (0.17–0.20) than that in montane populations (0.05–0.10; Table 2). Genes that were presumably in the same linkage block as outlier loci (within 5 kb in either direction) were annotated (a total of 73 candidate genes; Supporting information Table S1).

**Table 2:** Observed (*H*_o_) and expected (*H*_e_) heterozygosity in 525,866 neutral loci and 435 outlier loci

Population	Ecotype	Neutral		Outlier	
		*H*_o_	*H*_e_	*H*_o_	*H*_e_
Little Jacks	Desert	0.21	0.23	0.16	0.20
Big Jacks	Desert	0.17	0.21	0.10	0.17
Keithley	Cool montane	0.17	0.21	0.05	0.07
Johnson	Cool montane	0.18	0.22	0.04	0.06
Upper Mann	Cold montane	0.19	0.21	0.04	0.05
Fawn	Cold montane	0.16	0.20	0.07	0.10

### 3.4 Neutral and adaptive population structure

**Figure 4:**
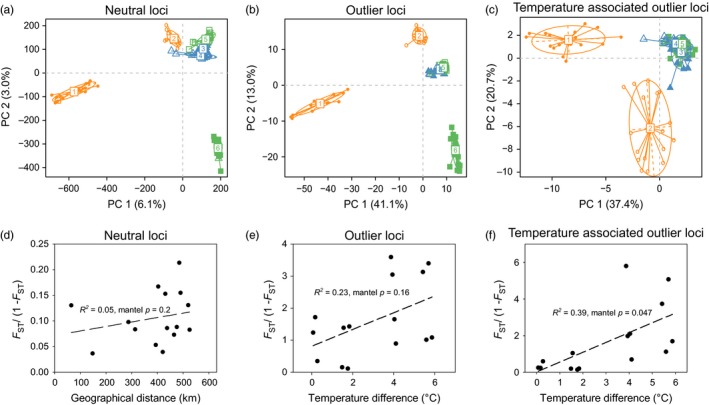
Population structure of six redband trout populations. Principal component analysis demonstrated population structure using 525,866 neutral loci (a), 435 outlier loci (b), and 20 outlier loci that were associated with summer temperature (c). Yellow color points: desert ecotype; blue: cool montane ecotype; green: cold montane ecotype. 1: Little Jacks; 2: Big Jacks; 3: Keithley; 4: Johnson; 5: Upper Mann; 6: Fawn. Panel d shows the pattern of isolation by distance using all neutral loci and fluvial distance among populations. Patterns of isolation by maximum stream temperature in summer (July and August 2015) were analyzed using all outlier loci (e) and outlier loci that are associated with summer temperatures (f)

Population structure was investigated by PCA using neutral (525,866 SNPs) and adaptive loci (435 outlier SNPs) (Figure 4a,b). In this study system, the two most isolated populations are also from the most extreme warm and extreme cold environments relative to the other four populations, which results in similar patterns in neutral and adaptive loci PCA, but due to different evolutionary processes. In neutral PCA, Little Jacks and Fawn are more differentiated from all others due to genetic drift in relatively isolated locations. In adaptive PCA, populations from the warmest and coldest streams were the most distinct from all others, reflecting signals of local adaptation. Interestingly, populations from the same ecotype (desert and cold montane streams) were not necessarily clustered together suggesting the potential for independent adaptation. This is also true for the cold montane populations. Further screening for temperature‐related outliers was conducted by association analyses between minor allele frequency and mean summer stream temperature for each outlier. The 20 markers most significantly associated with temperature (<5% of all outliers) were used to reconstruct the PCA plot, which clearly separated the desert and montane populations (Figure 4c). Interestingly, fish from the two desert streams (Little Jacks and Big Jacks) remained distinct, suggesting they may have evolved with alternative strategies for adaptation to warm environments, or faced different additional selective pressures. Annotation of the temperature associated outlier loci identified candidate genes NECP1, PKHM2, CRA1A, IRX5, NOS, GPR17 and GSTT1 (Supporting information Table S1). According to the neutral SNPs, isolation by distance model is not significantly supported (Figure 4d). A model of isolation by temperature was not significant when considering all outlier loci (Figure 4e), but it was significant when using the temperature associated outlier loci (Figure 4f).

### 3.5 Genetic basis of adaptive traits

**Figure 5:**
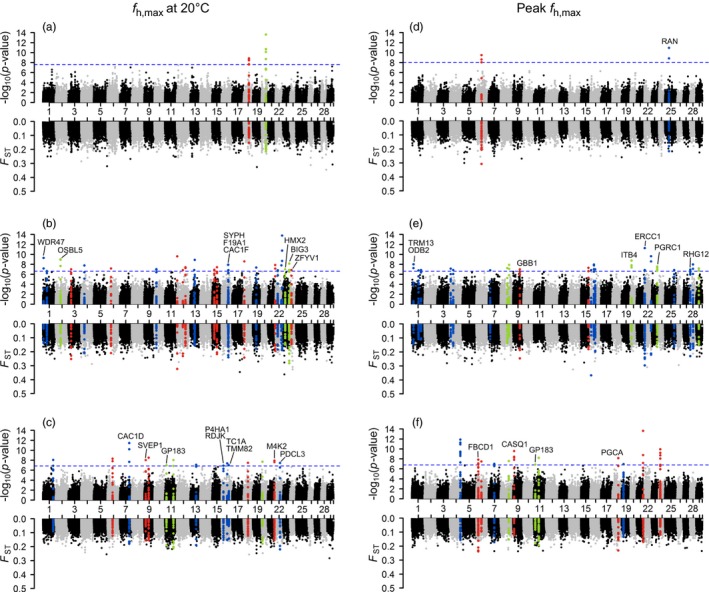
*F*_ST_ between cardiac phenotype classes at each locus within each ecotype. The *x*‐axis is the marker position for chromosome number 1–29. Markers that were not assigned a chromosome position are placed as chromosome 30. Phenotypes of *f*_h,max_ at 20°C (a‐c) and peak *f*_h,max_ (d‐f) were evenly divided into two classes (low and high) within each ecotype (desert = a and d, cool montane = b and e, cold montane = c and f). *F*_ST_ was calculated for each locus between the two phenotypic classes for a sliding window size of 10 kb (step size 200 bp). Fisher's exact test was used to test the significance for each window. Dashed blue line represents the threshold after BY‐FDR. Color points represent the sliding windows within 50 kb of the significant loci. Mapping details and annotation of significant sliding windows can be found in Supporting information Table S2

**Figure 6:**
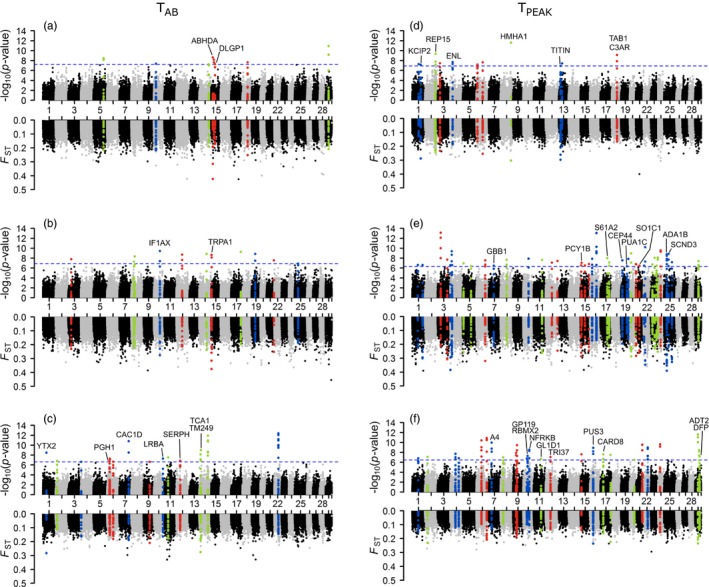
*F*_ST_ between phenotypic classes of thermal tolerance at each locus. The *x*‐axis is the marker position for chromosome number 1–29. Markers that were not assigned a chromosome position are placed as chromosome 30. Phenotypes of Arrhenius breakpoint point (*T*_AB_) (a‐c) and temperature of peak *f*_h,max_ (*T*_PEAK_) (d–f) were evenly divided into two classes (low and high) within each ecotype (desert = a and d, cool montane = b and e, cold montane = c and f). *F*_ST_ was calculated for each locus between the two phenotypic classes for a sliding window size of 10 kb (step size 200 bp). Fisher's exact test was used to test the significance for each window. Dashed blue line represents the threshold after BY‐FDR. Color points represent the sliding windows within 50 kb of the significant loci. Mapping details and annotation of significant sliding windows can be found in Supporting information Table S2

As we characterized cardiac response to acute thermal stress, it was also possible to explore the genetic basis of cardiac phenotypes (*f*_h,max_ at 20°C, peak *f*_h,max_, *T*_AB_, and *T*_PEAK_) within each ecotype. The *f*_h,max_ at 20°C was chosen not only because 20°C is close to *T*_AB_ but 20°C was also the highest temperature that almost all fish could maintain regular heartbeat. Results suggested candidate genomic regions associated with cardiac phenotypes within each ecotype including *f*_h,max_ at 20°C (47 regions in 20 chromosomes; Figure 5a–c), peak *f*_h,max_ (39 regions in 19 chromosomes; Figure 5d–f), *T*_AB_ (35 regions in 21 chromosomes; Figure 6a–c), and *T*_PEAK_ (86 regions in 25 chromosomes; Figure 6d–f). More candidate regions were identified in cold (36.2%) and cool (52.2%) montane ecotypes than desert populations (11.6%). Annotation of these significant genomic regions identified 62 unique candidate genes (Supporting information Table S2), which included ion channels (CAC1F, CAC1D, CASQ1, KCIP2, and TRPA1), muscle component (TITIN), and stress responses (SERPH).

## 4 DISCUSSION

To study evolutionary mechanisms of thermal adaptation, it is essential to integrate comparative physiology and population genomics to achieve broad understanding of processes involved. Compared to conventional studies under laboratory conditions (i.e., common garden environment (Chen et al., 2018)), field study has the potential to offer more ecologically relevant results and can better reflect thermal requirements of local environments. In this study, we extended results from a previous common garden design to field studies with a portable ECG apparatus to estimate cardiorespiratory functions for natural populations under the hypothesis of oxygen‐ and capacity‐limited thermal tolerance. Evidence of selection on cardiac function among ecotypes of redband trout was consistent with common garden results (Chen et al., 2018), and dense genome coverage with 526,301 SNPs provided the most detailed understanding to date in the candidate genes that are associated with thermally adaptive traits.

### 4.1 Intraspecific variation in cardiac function

Physiological experiments in field conditions are challenging, but recent studies illustrate that this can be a powerful approach to advance knowledge of local adaptation. A notable example is the study of sockeye salmon adaptation to spawning migration temperatures in Fraser River in British Columbia, Canada (Eliason et al., 2011). The authors used a portable respirometer to characterize cardiorespiratory function in natural populations and present functional divergence in aerobic scope, cardiac function, and adrenoreceptor density. In the present study, *f*_h,max_ results also showed an intraspecific pattern that was associated with summer stream temperatures in ecologically divergent populations of redband trout. The desert ecotype fish that have evolved under the warmest water temperatures demonstrated the best cardiac performance relative to montane fish as estimated with *f*_h,max_ across populations and therefore improved capacity to deliver oxygen to internal tissues under thermal stress. Acclimatization in the field can play a significant role in physiological phenotypes and affect performance. In present study, fish were sampled from each stream before river temperatures exceeded thermal optima. Sampling efforts occurred at each location with stream temperatures ranging from 11.6 to 18.8°C at time of fish capture, which is within the optimum thermal range for redband trout (Chen et al., 2018; Gamperl et al., 2002; Meyer et al., 2010). This pattern of *f*_h,max_ variation among ecotypes was also consistent with our previous study that used three of the same populations in this study but acclimated under a common garden condition (Chen et al., 2018). According to the OCLTT hypothesis, a higher heart rate allows fish to better maintain cardiac output and oxygen supply to tissues for activities under hot summer conditions. Additionally, *T*_AB_ was also higher in populations from warmer ecotypes, which represents the first rate transition temperature for *f*_h,max_ and has been shown to be close to the thermal optimum for aerobic scope in multiple species (Casselman et al., 2012). Furthermore, desert redband trout have been shown to have a broader thermal optimum window that extends to a much higher temperature than other ecotypes (Chen et al., 2018). Therefore, results from common garden and field studies both demonstrated similar intraspecific pattern: redband trout that evolved in extreme desert environments have improved thermal performance of cardiac function. More broadly, results from this study also support patterns of improved thermal tolerance in a domesticated strains of rainbow trout that has undergone thermal selection (Chen et al., 2015).

The *T*_PEAK_ represents the upper limits for activities that require the heart to work at its maximum rate, which is a rare but sometimes necessary and life‐saving event. All populations had the same *T*_PEAK_ at around 23°C, suggesting either critical temperature of maximum cardiac function is not under strong natural selection, or there are constraints limiting the selection on upper critical temperatures. Constraints may include lower critical temperatures in winter because critical low and high thermal tolerance have been found to change in parallel (Fangue, Hofmeister, & Schulte, 2006). Redband trout has a critical thermal maximum (CT_MAX_) around 28–30°C (Chen et al., 2018; Rodnick et al., 2004) and on average, *T*_PEAK_ was 5–6°C below CT_MAX_. Note that *f*_h,max_ is pharmacologically stimulated without any external control while CT_MAX_ is measured in conscious animals. Under acute situations a conscious fish has control of cholinergic inhibition on heart rate (Ekström, Hellgren, Gräns, Pichaud, & Sandblom, 2016), and maximum cardiac capacity can be reserved for higher temperatures. Indeed, measurement of the heart rate in conscious trout rarely exceeds 120 bpm, independent of body size and acclimation conditions (Aho & Vornanen, 2001; Altimiras & Larsen, 2000). Fish could also reduce their oxygen consumption through limiting their activities, for example, suppression of feeding and digestion under stress conditions. Despite that, *T*_PEAK_ of Little Jacks and Big Jacks trout are ~3°C below their maximum summer temperature. Therefore, fish from desert climates have risk of exposure to temperatures that are above the cardiac limits based on historic recorded high temperatures (Zoellick, 1999). Still to be investigated, however, is the plasticity of heart rate in redband trout during thermal acclimation.

### 4.2 Putative genomic signatures of thermal adaptation

Although genetic differentiation among populations is a result of both neutral and adaptive evolution, only variation at adaptive loci provides potential for selection to drive evolutionary adaptation to local environments. For both neutral and outlier loci, we observed a deficit of heterozygotes, which was not likely due to Wahlund effect because heterozygosity was calculated for individuals from the same population. In our study with dense genome coverage for a polygenic trait such as thermal tolerance, some adaptive SNPs would be expected to be out of Hardy–Weinberg equilibrium resulting in overall heterozygote deficits, especially when selection occurred in the sampled generation. We also acknowledge that some null alleles may exist in the dataset due to the nature of RAD‐seq, which may result in systematic underestimation of polymorphism and cause reduction in heterozygous genotypes (Arnold, Corbett‐Detig, Hartl, & Bomblies, 2013; Davey et al., 2013). Genotyping error such as heterozygotes miscalled as homozygotes could also be a result of the library construction protocol and bioinformatic pipeline filtering (Andrews, Good, Miller, Luikart, & Hohenlohe, 2016; Shafer et al., 2017). In this study, the majority of markers had similar observed and expected homozygosity, but a proportion of markers might have been affected by these factors (Supporting information Figure S2) and contribute to the heterozygote deficit. Heterozygote deficits were observed for both neutral and outlier loci; however, deficits were more common in outlier loci (Supporting information Figure S3) suggesting a role of selection. According to PCA results for neutral loci, the two most distinct populations in this study were from the most extreme warm and cold streams, reflecting genetic drift in relative isolation from other populations. Interestingly, adaptive outlier loci had nearly identical cluster patterns with the most distinct signal of adaptation occurring in populations from extreme desert and cold montane environments, which suggests evolutionary adaptation in isolated populations. However, populations of the same ecotype did not cluster together with outlier loci. One explanation is that populations have used different adaptive strategies and have evolved independently to the environmental conditions in relative isolation from one another. In addition, temperature may not be the only environmental factor driving selection. Other environmental factors might involve stream complexity, for example, thermal refugia, stream discharge, conductivity, dissolved oxygen, precipitation (Meyer et al., 2010; Muhlfeld, 2002; Narum et al., 2010). How much the difference in these factors has caused genetic divergence among populations is yet to be quantified. Another explanation could be related to confounding factors, such as geography or underlying genetic structure that could result in false positives. In this study, we included two populations from each ecotype to control the effect of confounding factors but we acknowledge that two replicate populations per ecotype are still limited to make general conclusions.

The subset of 20 outlier loci that were associated with summer stream temperature may reflect genomic regions specifically related to thermal adaptation. Candidate genes are involved in functions such as cardiac function (IRX5) (Costantini et al., 2005), development (CRA1A), and nervous system recovery (GPR17). Functions of most other genes are not well studied, especially in fish species, and demand further functional verification of their roles in thermal tolerance and performance.

### 4.3 Putative genomic regions underlie cardiac phenotypes

Like most phenotypic traits with a polygenic basis, thermal tolerance and performance are quantitative and the effect of genes ranges from small to medium. Genes with small allele frequency changes over generations are less likely to be identified by genome scans among populations (Hoban et al., 2016). Here, we further examined associations of markers across the genome with cardiac phenotypes within each ecotype. Candidate genes underlying phenotypic variation may not be directly under selection in populations that do not experience extreme temperatures, but warrant further verification in broadscale studies. Results here represent the first effort to explore the genetic basis of *f*_h,max_ and its thermal sensitivity in natural populations. For the four traits, we analyzed, two focused on *f*_h,max_ (*f*_h,max_ at 20°C and peak *f*_h,max_) and two focused on the thermal sensitivity of *f*_h,max_ (*T*_AB_ and *T*_peak_). The *f*_h,max_ at 20°C and peak *f*_h,max_ were inter‐related at the individual level (Supporting information Figure S1). Peak *f*_h,max_ has been found to have an additive genetic basis in salmonids (Chen et al., 2018; Muñoz et al., 2015). Difference in *f*_h,max_ could be related to the various membrane‐bound ion pumps, ion exchangers, and voltage‐gated ion channels involved in pacemaker action potential, which determines the intrinsic rhythm of the heartbeat. In the present study, we found that genetic variation in some Ca^2+^‐handling protein genes (e.g., CAC1D, CASQ1, and CAC1F) was associated with the *f*_h,max_ at 20°C. The molecular mechanism for rate limiting temperatures of *f*_h,max_ is still unknown. It may involve cellular functions within the ventricle, such as ion channel and energy supply, but not in pacemaker cells (Haverinen, Abramochkin, Kamkin, & Vornanen, 2017). A previous study also showed an additive genetic basis for *T*_PEAK_, but not *T*_AB_ (Muñoz et al., 2015). This study explored the genetic basis for both traits and identified candidate genes. Candidate genes for *T*_AB_ include CAC1D, which has been aforementioned as a candidate for *f*_h,max_, and a 47‐kda heat‐shock protein (SERPINH1), a chaperone in the biosynthetic pathway of collagen. Some candidate genes for *T*_PEAK_ are well‐known for their roles in cardiac function. For example, TITIN is involved in passive tension and plays a key role in contraction of muscle cells (Keen, Klaiman, Shiels, & Gillis, 2017). ADA1B (alpha 1B‐adrenergic receptor) has been shown to be involved in cardiac hypertrophy (Milano et al., 1994) and the regulation of vasoconstriction in mammals (Adefurin et al., 2017). The list of candidate genomic regions and genes generated here are presumably involved in thermal adaptation and need further functional verification. There is evidence across studies supporting candidate loci from heat‐shock genes, especially in the HSP40 family (Supporting information Table S3) (Chen et al., 2018; Narum et al., 2010; Narum, Campbell et al., 2013). However, each study applied distinct sets of markers because of different genotyping methods to address specific questions. Future projects with whole genome resequencing may reach a high marker density (over one million markers) to overcome some of the limitations of comparing marker sets with greater overlap.

Some limitations in this study could be considered in future studies, such as genome coverage and sample sizes. Thermal performance is polygenic as shown from the present and previous studies (Everett & Seeb, 2014; Jackson et al., 1998; Muñoz et al., 2015), and future studies may require whole genome resequencing to pinpoint specific genomic regions and variants associated with thermal tolerance. Although we used a dense reduced representation method (*Pst‐*I RAD‐seq), this approach covered only ~10% of the genome and other methods that cover 50% or more may be necessary. Furthermore, epigenetic factors, for example, DNA methylation (Le Luyer et al., 2017), may also be an important mechanism in evolutionary thermal adaptation and warrant investigation. Finally, while a larger sample size for each population would improve the statistical power to detect phenotypic divergence, our cardiac phenotyping method is time‐consuming and necessarily constrains the desire to achieve a large sample size. The number of populations we sampled for each ecotype is largely limited by the same reason but will be taken into consideration in future study designs.

## 5 CONCLUSIONS

This study integrated comparative physiology and population genomics to address the evolution of an aquatic ectotherm to local environments and explored mechanisms involved with thermal adaptation. We demonstrated differentiation of cardiac functions among natural populations from distinct thermal ecotypes, supporting the hypothesis of a selection for oxygen‐ and capacity‐related thermal performance and tolerance (Farrell, 2009; Pörtner et al., 2017). Additionally, we showed patterns of evolutionary adaptation to extreme climates at the genomic level and identified potential candidate genes. By investigating intraspecific divergence at multiple levels with cross‐discipline collaboration, results from this study can help develop connections between environment, functional adaptation, and genomic differentiation. This approach may be critical for understanding evolutionary potential of natural populations that may be threatened by changing climates.

## ACKNOWLEDGEMENTS

This work was supported by the Bonneville Power Administration through grant 200900500. APF is supported by the Natural Sciences and Engineering Research Council of Canada and holds a Canada Research Chair. The authors thank J. Stephenson with field sampling and temperature loggers.

## CONFLICT OF INTEREST

None declared.

## DATA ARCHIVING STATEMENT

Data available from the Dryad Digital Repository: https://doi.org/10.5061/dryad.kf674kb. RAD‐tag sequences are available from NCBI SRA database with accession number of SRP151085.
