# Supplementary figures and images for: Physiological and genomic signatures of evolutionary thermal adaptation in redband trout from extreme climates
Source: Evol Appl. 2018 Jul 20;11(9):1686–99. doi: 10.1111/eva.12672 (PMC6183465; doi:10.1111/eva.12672)

**Figure S1** Correlation among cardiac phenotypes across all populations.


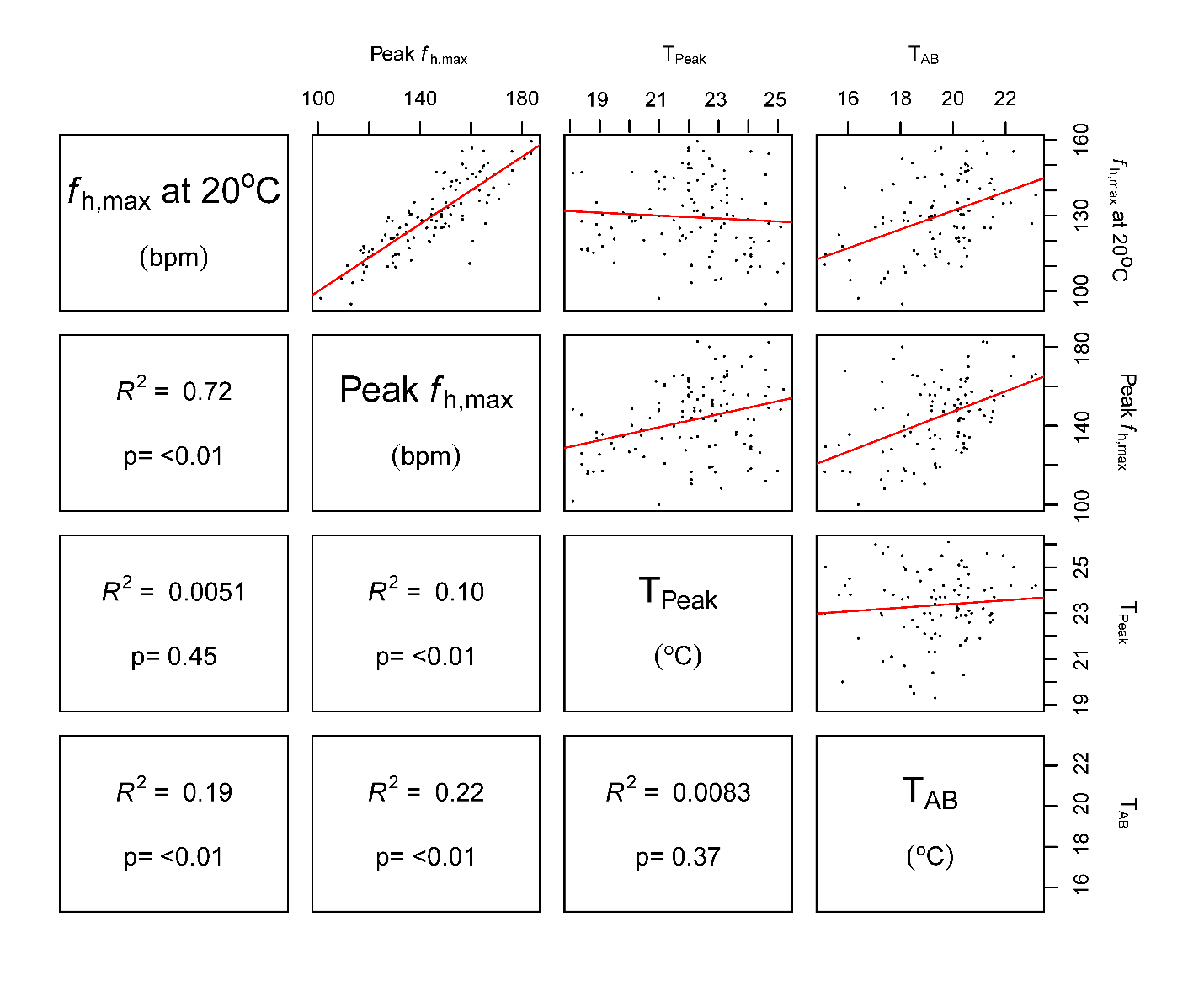


|  |  |  |
| --- | --- | --- |

Supplement: Supplementary file 1 [file EVA-11-1686-s001.docx]
