# Supplementary material for: Physiological and genomic signatures of evolutionary thermal adaptation in redband trout from extreme climates
Source: Evol Appl. 2018 Jul 20;11(9):1686–99. doi: 10.1111/eva.12672 (PMC6183465; doi:10.1111/eva.12672)
Supplement: Supplementary file 2 [file EVA-11-1686-s002.docx]

**Figure S2** Observed and expected frequency of each genotype (AA: homozygous for the reference allele; Aa: heterozygote; aa: homozygous for the alternate allele) across 526,301 markers.

| 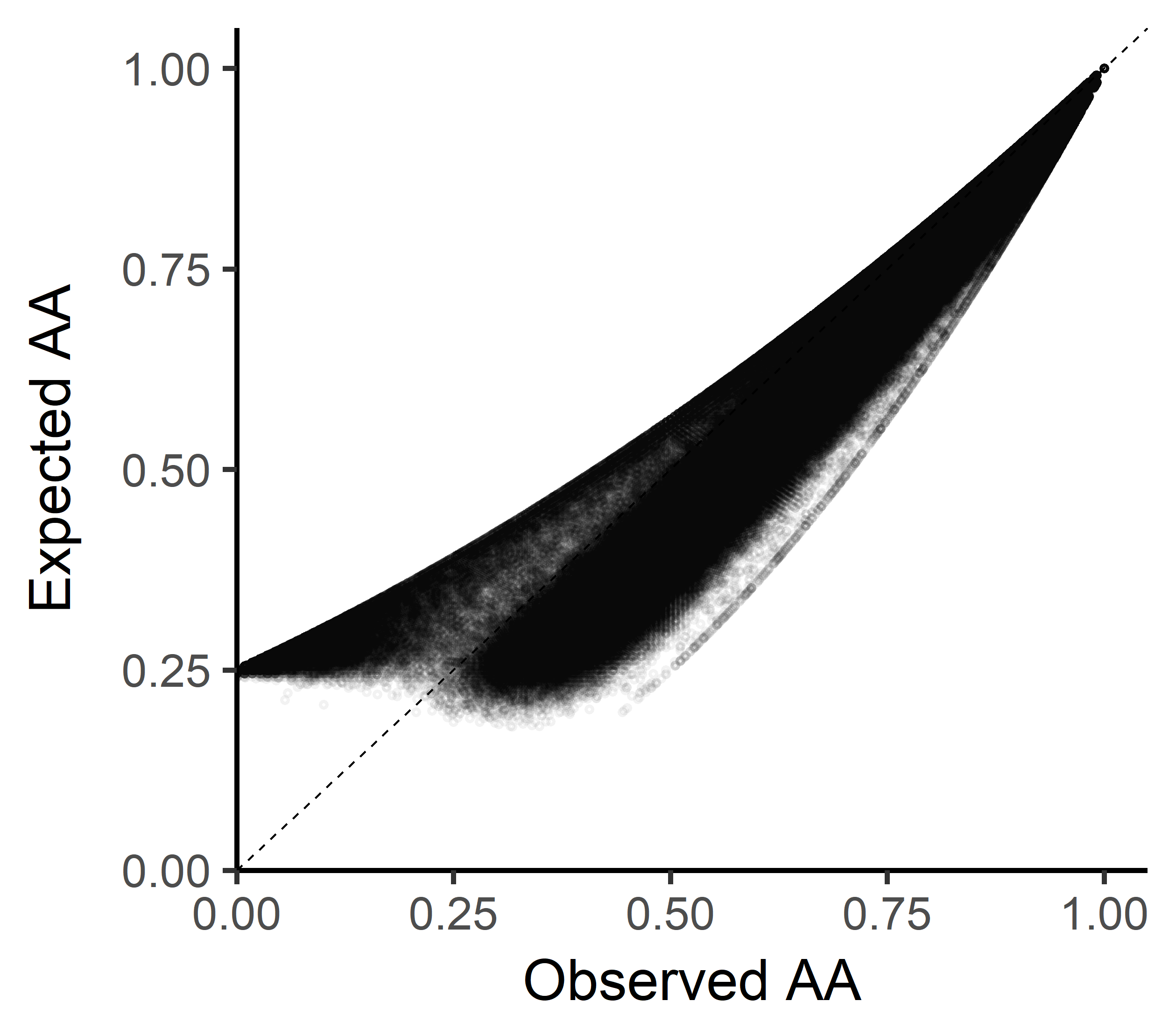 | 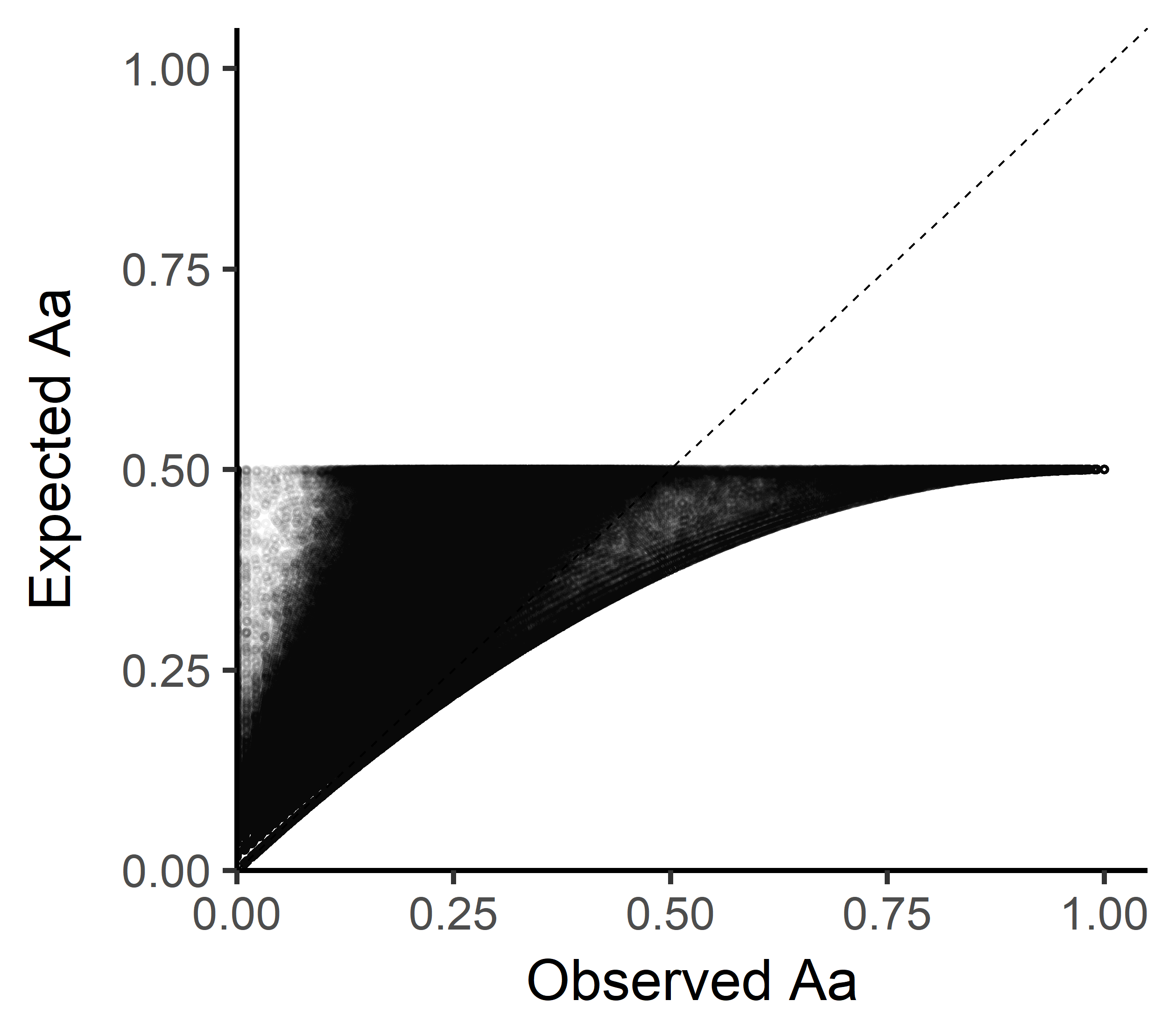 | 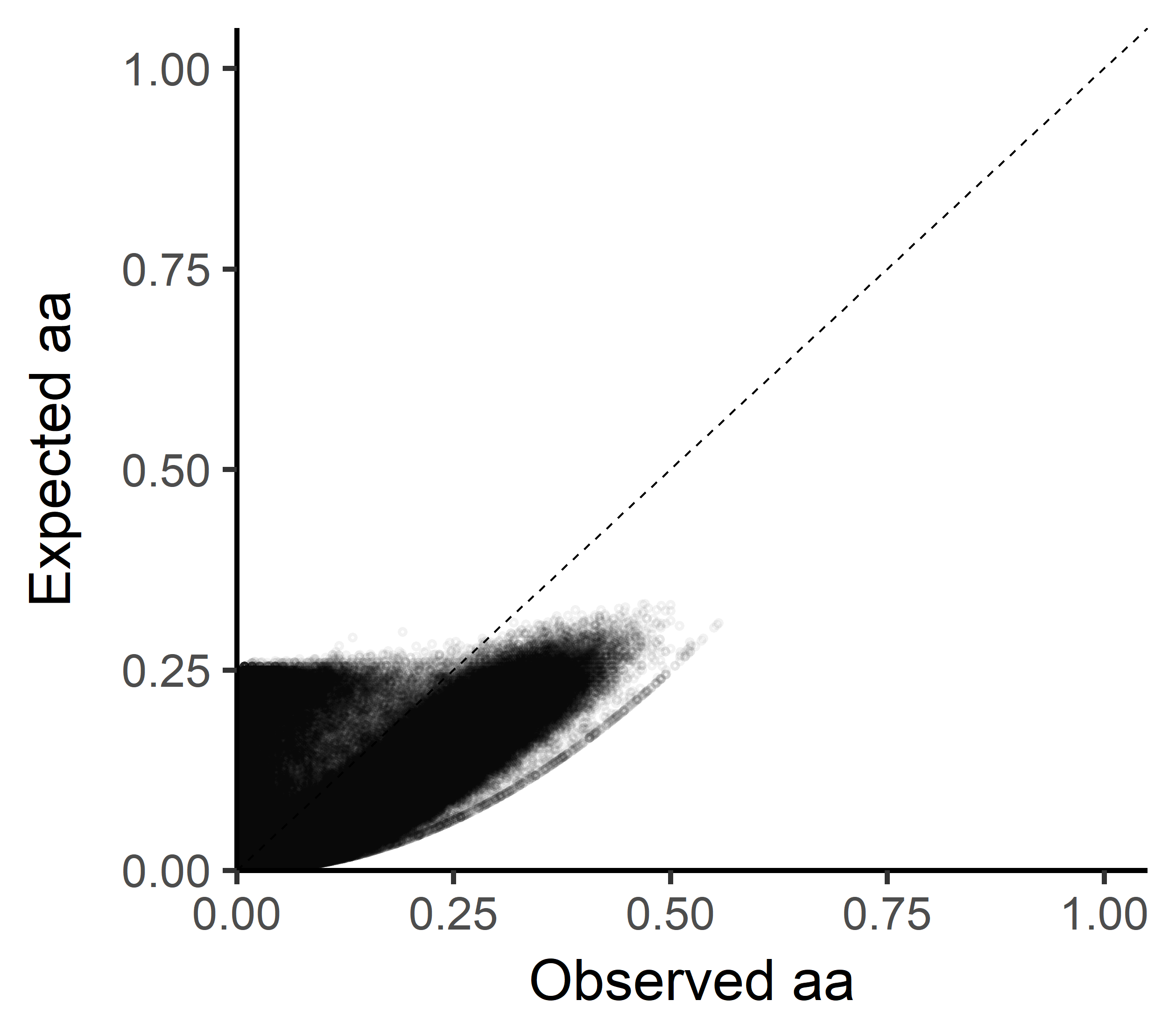 |
| --- | --- | --- |
