# Supplementary material for: Physiological and genomic signatures of evolutionary thermal adaptation in redband trout from extreme climates
Source: Evol Appl. 2018 Jul 20;11(9):1686–99. doi: 10.1111/eva.12672 (PMC6183465; doi:10.1111/eva.12672)
Supplement: Supplementary file 3 [file EVA-11-1686-s003.docx]

**Figure S3** Observed and expected homozygosity (1-hetezygosity) for neutral and outlier loci.

| **All populations - Neutral loci** | |
| --- | --- |
| 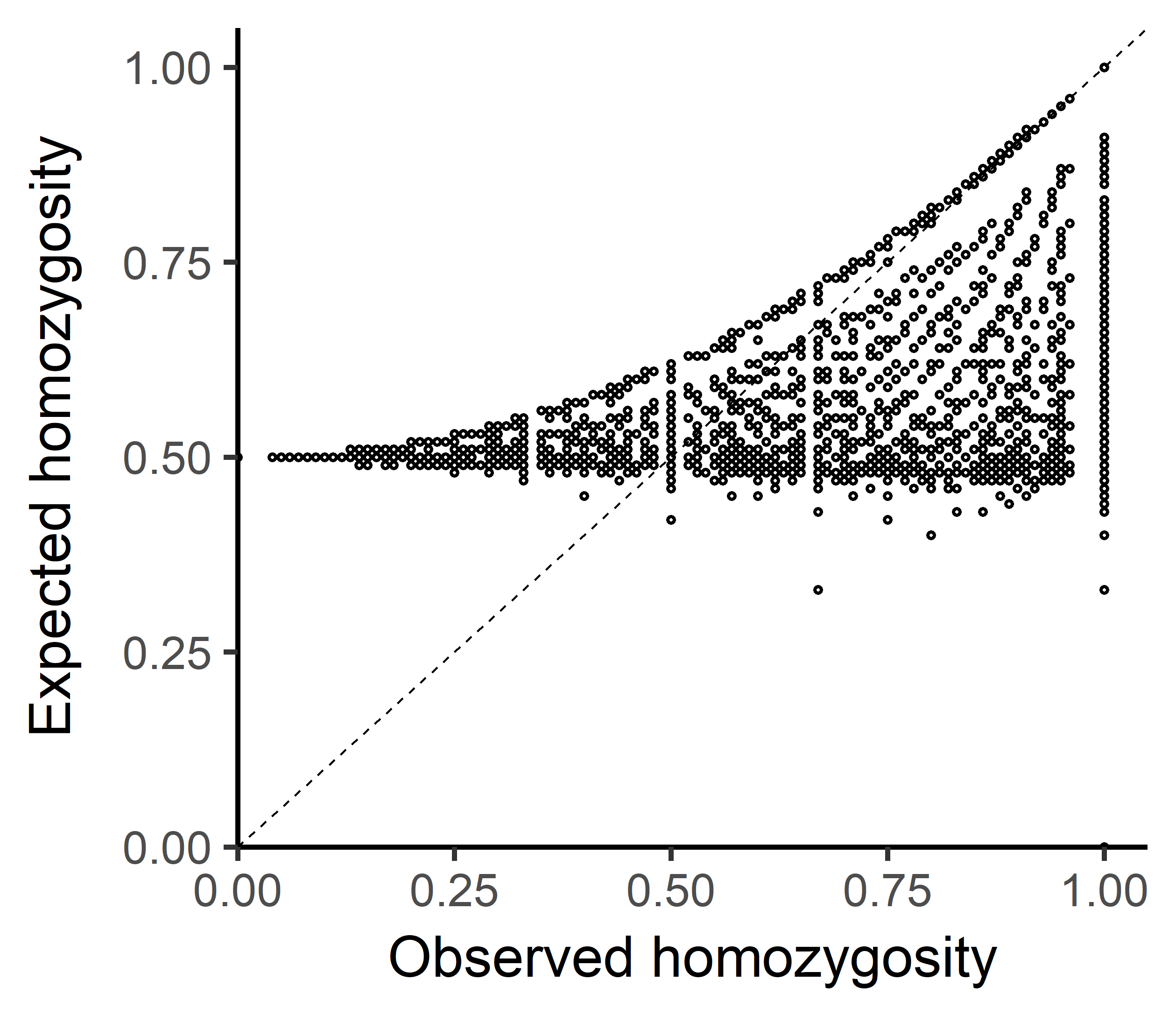 | 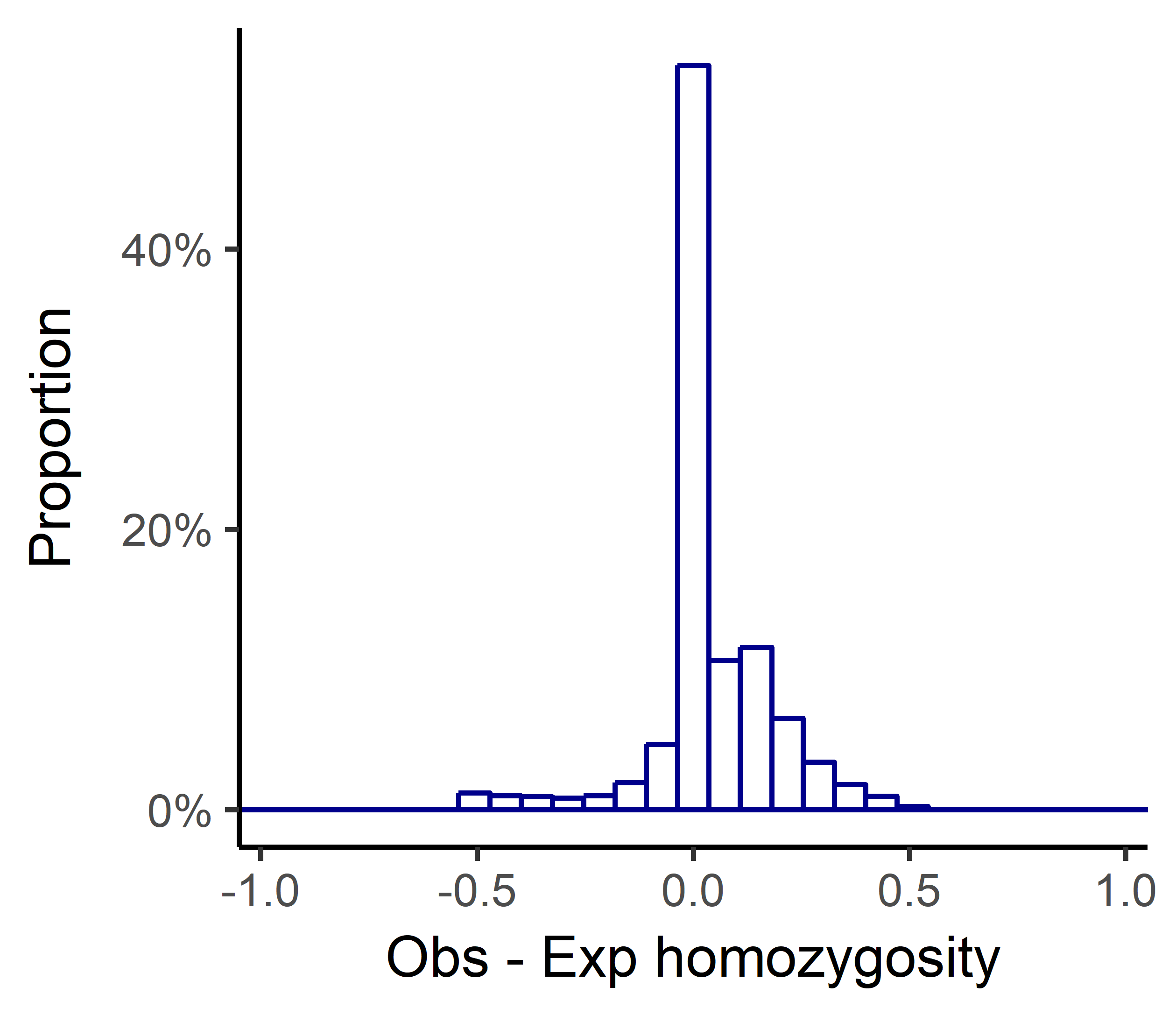 |

| **All populations - Outlier loci** | |
| --- | --- |
| 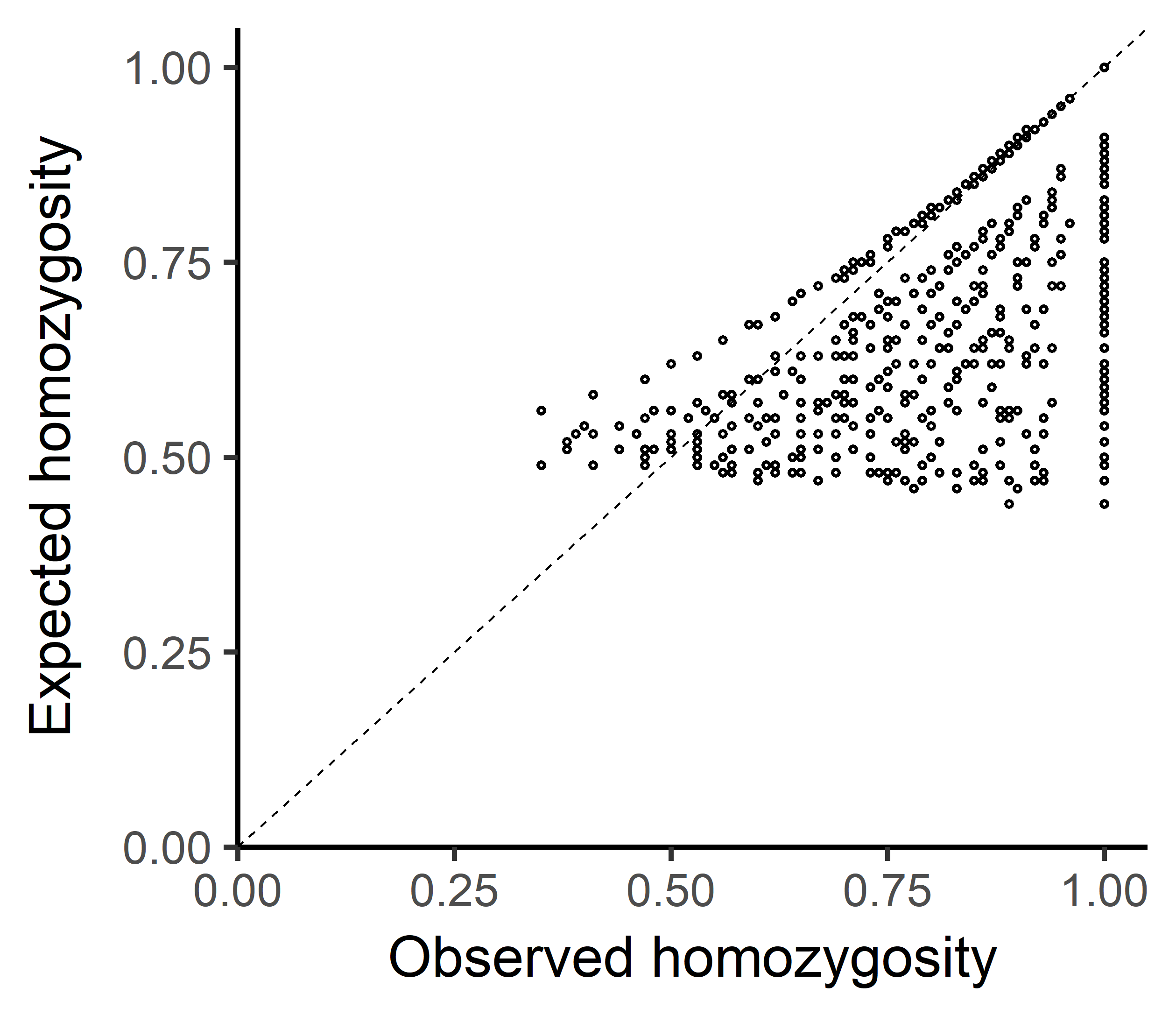 | 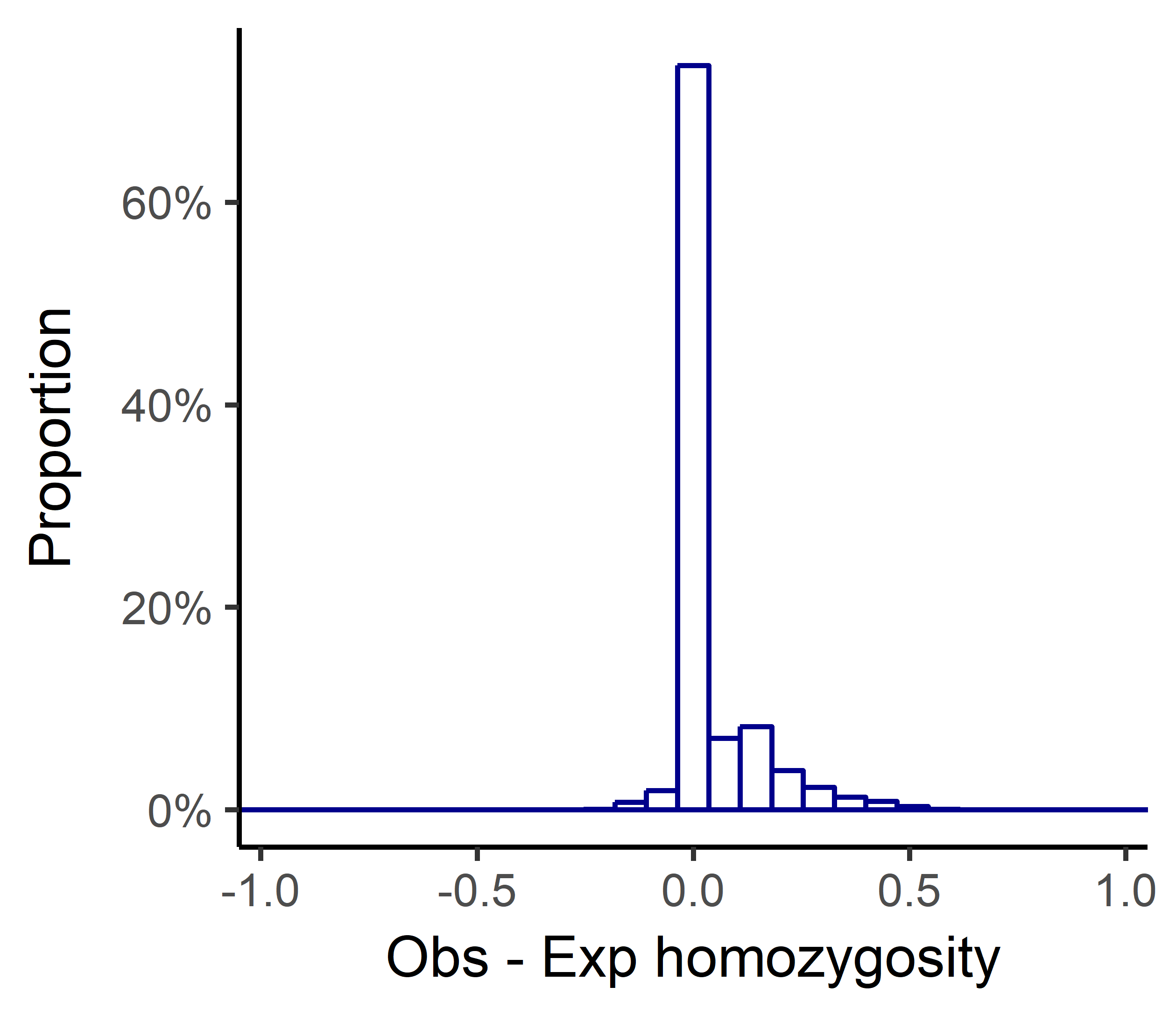 |
