# Supplementary material for: Physiological and genomic signatures of evolutionary thermal adaptation in redband trout from extreme climates
Source: Evol Appl. 2018 Jul 20;11(9):1686–99. doi: 10.1111/eva.12672 (PMC6183465; doi:10.1111/eva.12672)
Supplement: Supplementary file 4 [file EVA-11-1686-s004.pdf]

Table S1 Annotation of outlier loci.

Outlier	Chr	Chr_pos	Candidate gene (5 kb)	Environment Variable	p-value	R2	Allele_diff
957741_40	omy01	4044115		Summer temperature	0.357	0.28	0.74
462534_55	omy01	4228524	NR5A2_CHICK;	Summer temperature	0.254	0.40	1.00
502540_64	omy01	42976129		Summer temperature	0.030	0.83	0.93
688734_11	omy01	43109936		Summer temperature	0.057	0.75	0.89
1235980_40	omy01	43119933		Summer temperature	0.111	0.63	1.00
815286_13	omy01	44172181	IRX5_MOUSE;	Summer temperature	0.029	0.84	0.83
315622_38	omy01	45704573		Summer temperature	0.294	0.35	0.77
1273068_70	omy01	54004414		Summer temperature	0.178	0.51	0.95
889712_44	omy01	58633407		Summer temperature	0.341	0.30	0.75
715269_22	omy02	23946227	MBOA5_RAT;	Summer temperature	0.150	0.55	1.00
1251715_11	omy02	32957825		Summer temperature	0.053	0.76	1.00
1208243_74	omy02	36611582		Summer temperature	0.046	0.78	0.81
695584_26	omy02	39565560	RTXE_DROME;	Summer temperature	0.357	0.28	0.87
1248723_21	omy02	39638851		Summer temperature	0.392	0.25	0.95
738251_62	omy02	39881722		Summer temperature	0.389	0.25	0.88
489977_49	omy02	39926707		Summer temperature	0.406	0.24	0.91
704721_51	omy02	39947579		Summer temperature	0.342	0.30	0.95
704721_48	omy02	39947579		Summer temperature	0.342	0.30	0.95
332424_13	omy02	41516296		Summer temperature	0.424	0.22	0.90
355877_48	omy02	45340302		Summer temperature	0.210	0.46	1.00
593312_21	omy02	47922866		Summer temperature	0.335	0.30	0.82
371915_33	omy02	49596054		Summer temperature	0.366	0.27	0.94
416990_70	omy02	50791681	ANLN_XENLA;	Summer temperature	0.356	0.28	0.94
177716_8	omy02	51610079		Summer temperature	0.312	0.33	0.92
766033_44	omy02	62594530		Summer temperature	0.312	0.33	1.00
1039547_44	omy02	70940519		Summer temperature	0.107	0.63	1.00
510763_23	omy02	80094964		Summer temperature	0.357	0.28	0.71
689461_14	omy02	80485643		Summer temperature	0.357	0.28	0.86
1101676_48	omy02	82961170		Summer temperature	0.357	0.28	0.85
575808_57	omy03	27945468		Summer temperature	0.030	0.83	0.78
633401_11	omy03	72482646		Summer temperature	0.831	0.02	0.73
633401_12	omy03	72482646		Summer temperature	0.831	0.02	0.73
836509_22	omy03	77385369		Summer temperature	0.172	0.52	1.00
836509_71	omy03	77385369		Summer temperature	0.231	0.43	0.93
1408323_36	omy03	79883950		Summer temperature	0.429	0.22	0.92
1229224_45	omy03	84202266		Summer temperature	0.425	0.22	0.85
523026_21	omy04	1833076	TRI25_HUMAN;	Summer temperature	0.157	0.54	0.93
216197_62	omy04	9822010		Summer temperature	0.120	0.61	1.00
743803_72	omy04	16355399		Summer temperature	0.091	0.67	1.00
743803_73	omy04	16355399		Summer temperature	0.091	0.67	1.00
254757_59	omy04	28317023		Summer temperature	0.357	0.28	0.82
679393_40	omy04	34605832		Summer temperature	0.289	0.35	0.95
103064_59	omy04	35553700		Summer temperature	0.173	0.51	1.00
401414_49	omy04	36991814		Summer temperature	0.019	0.88	0.91

Outlier	Chr	Chr_pos	Candidate gene (5 kb)	Environment Variable	p-value	R2	Allele_diff
401414_73	omy04	36991814		Summer temperature	0.019	0.88	0.91
557041_61	omy04	44179816		Summer temperature	0.357	0.28	0.73
1287826_8	omy04	44849446		Summer temperature	0.357	0.28	0.77
1129791_51	omy04	48111472		Summer temperature	0.349	0.29	0.87
916146_31	omy04	48111543		Summer temperature	0.350	0.29	0.91
1291212_73	omy04	53529817	KLF11_MOUSE;	Summer temperature	0.338	0.30	0.90
277587_55	omy04	64756914	FAXC_XENTR;	Summer temperature	0.243	0.41	0.94
881628_60	omy04	68169866		Summer temperature	0.223	0.44	0.94
350721_53	omy04	68188765		Summer temperature	0.235	0.42	0.94
1341292_60	omy04	72252509		Summer temperature	0.323	0.32	1.00
799503_25	omy04	72411485		Summer temperature	0.381	0.26	0.83
118667_52	omy05	4121661		Summer temperature	0.270	0.38	1.00
397770_13	omy05	21018003		Summer temperature	0.062	0.74	0.93
942120_42	omy05	25653447		Summer temperature	0.336	0.30	1.00
380144_69	omy05	37908771	CLIP1_CHICK;	Summer temperature	0.144	0.56	0.95
831445_12	omy05	42552455	S27A4_MACFA;	Summer temperature	0.270	0.38	0.93
1254520_71	omy05	43359039		Summer temperature	0.390	0.25	0.89
1254520_74	omy05	43359039		Summer temperature	0.390	0.25	0.89
660016_57	omy05	45879032	CRA1A_DANRE;	Summer temperature	0.022	0.86	0.90
188762_37	omy05	49028926		Summer temperature	0.268	0.38	0.88
406393_48	omy05	49649886		Summer temperature	0.244	0.41	0.94
71649_27	omy05	49776110		Summer temperature	0.258	0.39	0.88
915061_9	omy05	49798638		Summer temperature	0.305	0.34	0.86
522988_63	omy05	52988055	PTBP1_PIG;	Summer temperature	0.252	0.40	1.00
848843_69	omy05	53025657		Summer temperature	0.050	0.77	1.00
1018106_54	omy05	58285130	SCC4_XENTR;	Summer temperature	0.288	0.36	0.86
1157846_63	omy05	83220712		Summer temperature	0.257	0.39	0.88
1119251_60	omy05	85293115	TOP1_XENLA;	Summer temperature	0.392	0.25	0.86
39971_40	omy05	85647746		Summer temperature	0.357	0.28	0.87
215478_46	omy05	86962207		Summer temperature	0.375	0.26	0.88
1426088_8	omy05	90136102		Summer temperature	0.318	0.32	0.93
1426088_37	omy05	90136102		Summer temperature	0.318	0.32	0.93
205005_74	omy06	7061986		Summer temperature	0.288	0.36	0.85
396806_32	omy06	7861552		Summer temperature	0.396	0.25	0.87
774949_9	omy06	10260208		Summer temperature	0.357	0.28	0.91
119288_8	omy06	10308290		Summer temperature	0.357	0.28	0.94
704454_51	omy06	10388840		Summer temperature	0.357	0.28	0.83
88314_18	omy06	10582757		Summer temperature	0.307	0.34	0.86
710542_33	omy06	10744038		Summer temperature	0.357	0.28	0.95
746690_45	omy06	12108548		Summer temperature	0.336	0.30	0.83
487424_49	omy06	12142386		Summer temperature	0.349	0.29	0.83
487424_50	omy06	12142386		Summer temperature	0.349	0.29	0.83
350156_69	omy06	13534601		Summer temperature	0.332	0.31	0.76
350156_61	omy06	13534601		Summer temperature	0.332	0.31	0.76
1426409_7	omy06	14567442		Summer temperature	0.369	0.27	0.92
542209_53	omy06	29085232	SETBP_HUMAN;	Summer temperature	0.162	0.53	0.83

Outlier	Chr	Chr_pos	Candidate gene (5 kb)	Environment Variable	p-value	R2	Allele_diff
542209_11	omy06	29085232	SETBP_HUMAN;	Summer temperature	0.162	0.53	0.83
1256848_23	omy06	35516309		Summer temperature	0.024	0.86	1.00
1108238_38	omy06	44887051		Summer temperature	0.223	0.44	1.00
875605_41	omy06	52742547		Summer temperature	0.251	0.40	0.92
348463_74	omy06	67740231		Summer temperature	0.009	0.92	1.00
956537_23	omy06	70166187		Summer temperature	0.009	0.92	0.86
1324185_69	omy07	19602963		Summer temperature	0.381	0.26	0.89
999167_69	omy07	20063353		Summer temperature	0.357	0.28	0.83
543211_28	omy07	20124964		Summer temperature	0.365	0.27	0.86
1133292_43	omy07	20687594		Summer temperature	0.312	0.33	0.82
881182_53	omy07	20731114		Summer temperature	0.252	0.40	0.94
399473_17	omy07	20906248		Summer temperature	0.357	0.28	0.78
588890_48	omy07	50062942	KCNC4_HUMAN;	Summer temperature	0.298	0.34	1.00
534487_10	omy07	50319612	MEP50_PONAB;	Summer temperature	0.331	0.31	1.00
534487_8	omy07	50319612	MEP50_PONAB;	Summer temperature	0.331	0.31	1.00
93093_33	omy07	50320679	MEP50_PONAB;	Summer temperature	0.331	0.31	1.00
367067_40	omy08	317280	RTJK_DROFU;	Summer temperature	0.297	0.35	0.82
1064777_57	omy08	18818057	PLMN_MOUSE;	Summer temperature	0.315	0.33	0.94
576566_10	omy08	19009047		Summer temperature	0.307	0.33	0.88
909287_34	omy08	19111866		Summer temperature	0.223	0.44	0.88
258577_38	omy08	21936008		Summer temperature	0.341	0.30	1.00
237919_51	omy08	22049037		Summer temperature	0.339	0.30	0.80
1321892_18	omy08	27067013		Summer temperature	0.313	0.33	0.95
625694_35	omy08	27271191		Summer temperature	0.357	0.28	0.73
442753_29	omy08	59875983		Summer temperature	0.562	0.12	1.00
1042521_50	omy08	81362675	AGO3_DANRE;	Summer temperature	0.268	0.38	0.94
889586_67	omy09	8851736		Summer temperature	0.089	0.67	0.88
1426449_30	omy09	13795005		Summer temperature	0.004	0.96	0.90
968136_24	omy09	15897911		Summer temperature	0.289	0.36	1.00
1013109_7	omy09	19270747		Summer temperature	0.127	0.59	0.83
946675_9	omy09	19454966		Summer temperature	0.033	0.83	1.00
946675_23	omy09	19454966		Summer temperature	0.033	0.82	1.00
89285_47	omy09	19520057		Summer temperature	0.031	0.83	0.89
89285_32	omy09	19520057		Summer temperature	0.032	0.83	0.89
1279926_18	omy09	19541263		Summer temperature	0.045	0.79	1.00
882071_56	omy09	20023549		Summer temperature	0.339	0.30	0.87
285661_56	omy09	21140455		Summer temperature	0.063	0.74	0.80
293822_22	omy09	21949487		Summer temperature	0.306	0.34	0.88
1152869_63	omy09	21955496		Summer temperature	0.348	0.29	0.88
842577_51	omy09	22278139		Summer temperature	0.306	0.34	0.88
376877_12	omy09	22674820		Summer temperature	0.317	0.32	0.82
1087137_17	omy09	22862274		Summer temperature	0.312	0.33	0.92
1087137_11	omy09	22862274		Summer temperature	0.312	0.33	0.88
932179_15	omy09	22916807	STIM2_HUMAN;	Summer temperature	0.288	0.36	0.88
105825_49	omy09	23254572		Summer temperature	0.339	0.30	0.73
766382_65	omy09	30632114	ATX1_RAT;	Summer temperature	0.312	0.33	0.94

Outlier	Chr	Chr_pos	Candidate gene (5 kb)	Environment Variable	p-value	R2	Allele_diff
663794_61	omy09	35438680		Summer temperature	0.034	0.82	1.00
928978_46	omy09	35446609		Summer temperature	0.091	0.67	0.93
928978_40	omy09	35446609		Summer temperature	0.091	0.67	0.93
1302081_51	omy09	39030425		Summer temperature	0.348	0.29	0.87
184543_64	omy09	42890194		Summer temperature	0.362	0.28	0.85
1009396_14	omy09	52415125	ORML2_HUMAN;	Summer temperature	0.034	0.82	1.00
132163_66	omy09	66713142		Summer temperature	0.066	0.73	0.85
344249_55	omy10	6575898		Summer temperature	0.270	0.38	0.92
488986_24	omy10	6660371		Summer temperature	0.227	0.43	1.00
104080_45	omy10	8463921		Summer temperature	0.312	0.33	0.89
1012347_63	omy10	8938956		Summer temperature	0.070	0.72	1.00
975217_23	omy10	10719642		Summer temperature	0.218	0.45	0.00
33207_44	omy10	26169043		Summer temperature	0.331	0.31	1.00
189813_27	omy10	26537379	TIM22_RAT;	Summer temperature	0.075	0.71	1.00
399557_62	omy10	30594738		Summer temperature	0.312	0.33	0.89
630313_29	omy10	33150796	PLST_HUMAN;	Summer temperature	0.293	0.35	0.82
630313_30	omy10	33150796	PLST_HUMAN;	Summer temperature	0.293	0.35	0.82
1096957_73	omy10	34967850		Summer temperature	0.357	0.28	0.75
494855_14	omy10	35930634		Summer temperature	0.074	0.71	0.72
590544_8	omy10	35934453		Summer temperature	0.089	0.67	0.89
55326_44	omy10	40513690		Summer temperature	0.344	0.30	1.00
513234_42	omy10	41329805		Summer temperature	0.863	0.01	0.94
1015104_29	omy10	44073669		Summer temperature	0.441	0.21	0.95
1015104_61	omy10	44073669		Summer temperature	0.441	0.21	0.95
553363_52	omy10	46317415		Summer temperature	0.136	0.58	1.00
553363_19	omy10	46317415		Summer temperature	0.150	0.55	1.00
989212_32	omy10	47926715		Summer temperature	0.312	0.33	0.76
256264_29	omy10	48436980	ABCG4_HUMAN;	Summer temperature	0.382	0.26	0.88
624481_68	omy10	52647785		Summer temperature	0.260	0.39	0.94
59308_30	omy10	54805826		Summer temperature	0.345	0.29	0.93
1039822_24	omy10	55770155		Summer temperature	0.312	0.33	0.82
1039822_18	omy10	55770155		Summer temperature	0.312	0.33	0.87
601329_18	omy10	57111764		Summer temperature	0.331	0.31	0.87
1306584_69	omy11	1044105		Summer temperature	0.312	0.33	0.81
1340949_71	omy11	7247256		Summer temperature	0.266	0.38	0.93
965899_18	omy11	15845793		Summer temperature	0.367	0.27	0.90
1357762_31	omy11	28806494		Summer temperature	0.013	0.90	1.00
1357762_69	omy11	28806494		Summer temperature	0.013	0.90	1.00
800767_56	omy11	31005410		Summer temperature	0.093	0.66	0.94
560189_59	omy11	33429236		Summer temperature	0.085	0.68	1.00
1534217_39	omy11	37570389		Summer temperature	0.183	0.50	1.00
140792_14	omy11	37739571		Summer temperature	0.056	0.75	1.00
132289_71	omy11	37739642		Summer temperature	0.081	0.69	1.00
853708_23	omy11	42991302	GSTT1_BOVIN;	Summer temperature	0.030	0.83	1.00
1343968_7	omy11	45645377		Summer temperature	0.159	0.54	1.00
958797_19	omy11	46843377		Summer temperature	0.266	0.38	0.94

Outlier	Chr	Chr_pos	Candidate gene (5 kb)	Environment Variable	p-value	R2	Allele_diff
955104_64	omy11	47017227	LHX6_HUMAN;	Summer temperature	0.223	0.44	0.88
955104_57	omy11	47017227	LHX6_HUMAN;	Summer temperature	0.223	0.44	0.88
41048_48	omy11	47608147	LMO41_XENTR;	Summer temperature	0.267	0.38	0.87
41048_6	omy11	47608147	LMO41_XENTR;	Summer temperature	0.267	0.38	0.87
1002080_15	omy11	48549859		Summer temperature	0.051	0.77	1.00
450277_42	omy11	51552876		Summer temperature	0.357	0.28	0.83
563040_69	omy11	56752920		Summer temperature	0.037	0.81	0.88
683374_14	omy11	60428610	ATD1A_DANRE;	Summer temperature	0.337	0.30	0.23
808068_20	omy11	69076879		Summer temperature	0.283	0.36	0.88
285889_42	omy11	73805316		Summer temperature	0.250	0.40	0.88
1214983_17	omy12	24922179	UGT3_PLEPL;	Summer temperature	0.061	0.74	0.88
1124932_66	omy12	30922222		Summer temperature	0.288	0.36	0.88
559729_12	omy12	36373104		Summer temperature	0.312	0.33	0.76
1113153_25	omy12	36375168		Summer temperature	0.348	0.29	0.87
724650_5	omy12	36994111		Summer temperature	0.369	0.27	0.81
1050535_68	omy12	37147217		Summer temperature	0.495	0.17	0.94
1009423_15	omy12	38735050		Summer temperature	0.366	0.27	0.94
1101246_33	omy12	39853504		Summer temperature	0.343	0.30	0.88
1101246_37	omy12	39853504		Summer temperature	0.343	0.30	0.88
937912_60	omy12	40777630		Summer temperature	0.312	0.33	0.83
1114677_45	omy12	42735570		Summer temperature	0.341	0.30	0.94
1149671_33	omy12	42778784	POL3_DROME;	Summer temperature	0.299	0.34	1.00
1265613_31	omy12	42859678		Summer temperature	0.341	0.30	0.94
246529_72	omy12	43489509		Summer temperature	0.295	0.35	1.00
135973_52	omy12	58592726		Summer temperature	0.297	0.35	0.92
962826_13	omy12	59481516		Summer temperature	0.312	0.33	0.83
399357_14	omy12	59877844		Summer temperature	0.369	0.27	0.82
160213_51	omy12	62907678		Summer temperature	0.339	0.30	0.94
160213_32	omy12	62907678		Summer temperature	0.339	0.30	0.82
311377_42	omy12	69557088		Summer temperature	0.268	0.38	0.88
311377_65	omy12	69557088		Summer temperature	0.268	0.38	0.88
1340330_35	omy12	72036930		Summer temperature	0.303	0.34	0.89
1340330_6	omy12	72036930		Summer temperature	0.303	0.34	0.89
411070_30	omy12	73118561	NBR1_MOUSE;	Summer temperature	0.232	0.43	0.93
444883_37	omy13	2286603		Summer temperature	0.049	0.77	0.95
470874_34	omy13	17701663	APOL3_HUMAN;	Summer temperature	0.221	0.44	0.95
1046388_14	omy13	18511653		Summer temperature	0.141	0.57	1.00
488679_74	omy13	24129068		Summer temperature	0.163	0.53	0.95
488679_60	omy13	24129068		Summer temperature	0.163	0.53	0.95
579465_52	omy13	44310006		Summer temperature	0.151	0.55	1.00
1110689_48	omy13	48249247	HVM16_MOUSE;	Summer temperature	0.078	0.70	1.00
1269576_54	omy14	678680	KLH38_DANRE;	Summer temperature	0.312	0.33	0.88
289129_36	omy14	23095778		Summer temperature	0.441	0.21	0.83
289129_73	omy14	23095778		Summer temperature	0.458	0.19	0.83
176457_16	omy14	27550006	SEM6D_PONAB;	Summer temperature	0.109	0.63	1.00
1023339_44	omy14	30088251	NECP1_MOUSE;	Summer temperature	0.009	0.93	1.00

Outlier	Chr	Chr_pos	Candidate gene (5 kb)	Environment Variable	p-value	R2	Allele_diff
418329_63	omy14	31370031		Summer temperature	0.262	0.39	1.00
776275_64	omy14	33661544		Summer temperature	0.366	0.27	1.00
1168475_47	omy14	36571681		Summer temperature	0.270	0.38	1.00
180937_17	omy14	37881011		Summer temperature	0.252	0.40	0.93
952496_69	omy14	39795303	CERS2_HUMAN;	Summer temperature	0.273	0.37	1.00
863581_29	omy14	41414086		Summer temperature	0.248	0.41	0.94
649598_24	omy14	42382032		Summer temperature	0.261	0.39	1.00
352231_31	omy14	42382103		Summer temperature	0.261	0.39	1.00
357482_22	omy14	42513537		Summer temperature	0.280	0.37	1.00
107163_13	omy14	43513607		Summer temperature	0.237	0.42	0.93
1090652_11	omy14	43742288		Summer temperature	0.253	0.40	1.00
1090652_31	omy14	43742288		Summer temperature	0.253	0.40	1.00
991172_39	omy14	43800034		Summer temperature	0.234	0.42	0.94
814477_37	omy14	43872826		Summer temperature	0.251	0.40	1.00
814477_49	omy14	43872826		Summer temperature	0.251	0.40	1.00
1095928_54	omy14	43876815		Summer temperature	0.251	0.40	0.94
1327338_8	omy14	44000814	SLIK4_MOUSE;	Summer temperature	0.312	0.33	0.88
1113126_58	omy14	44173188		Summer temperature	0.258	0.39	0.94
459173_18	omy14	44262642		Summer temperature	0.245	0.41	0.93
312350_72	omy14	44319254		Summer temperature	0.268	0.38	1.00
312350_66	omy14	44319254		Summer temperature	0.268	0.38	1.00
361248_48	omy14	44503526		Summer temperature	0.325	0.32	0.93
793510_11	omy14	44507405		Summer temperature	0.263	0.39	0.93
793510_74	omy14	44507405		Summer temperature	0.266	0.38	0.93
1042314_48	omy14	44542472		Summer temperature	0.269	0.38	1.00
137536_60	omy14	44548687		Summer temperature	0.266	0.38	1.00
300943_14	omy14	44730005		Summer temperature	0.272	0.37	1.00
300943_13	omy14	44730005		Summer temperature	0.272	0.37	1.00
964188_63	omy14	44746595		Summer temperature	0.249	0.40	1.00
42582_34	omy14	44750874		Summer temperature	0.287	0.36	0.93
904942_22	omy14	45359496		Summer temperature	0.300	0.34	0.94
526596_44	omy14	45387390		Summer temperature	0.260	0.39	1.00
187502_42	omy14	46227448		Summer temperature	0.127	0.59	1.00
187502_63	omy14	46227448		Summer temperature	0.127	0.59	1.00
34284_27	omy14	46821864		Summer temperature	0.304	0.34	0.07
926290_31	omy14	47396831		Summer temperature	0.258	0.39	1.00
1158891_51	omy14	48328162		Summer temperature	0.261	0.39	1.00
1158891_52	omy14	48328162		Summer temperature	0.264	0.39	1.00
1310332_18	omy14	51807177	CD40L_CANFA;	Summer temperature	0.339	0.30	0.79
722873_18	omy14	60593229		Summer temperature	0.002	0.97	0.96
937809_58	omy14	64376611		Summer temperature	0.022	0.86	0.81
496358_46	omy14	66660217	RNSL3_DANRE;	Summer temperature	0.041	0.80	0.94
1130700_20	omy14	70497575		Summer temperature	0.084	0.68	0.93
1130700_23	omy14	70497575		Summer temperature	0.084	0.68	0.93
92976_45	omy14	72017078	STAG2_MOUSE;	Summer temperature	0.041	0.80	0.90
199616_43	omy14	73617841		Summer temperature	0.070	0.72	1.00

Outlier	Chr	Chr_pos	Candidate gene (5 kb)	Environment Variable	p-value	R2	Allele_diff
190858_55	omy14	77402388		Summer temperature	0.389	0.25	0.93
280703_56	omy14	79778356		Summer temperature	0.339	0.30	1.00
1136807_72	omy15	12691587		Summer temperature	0.331	0.31	0.85
1039753_45	omy15	13032108	NEBU_HUMAN;	Summer temperature	0.309	0.33	0.88
730580_6	omy15	13036784	NEBU_HUMAN;	Summer temperature	0.336	0.30	0.89
347904_60	omy15	14688894		Summer temperature	0.368	0.27	0.85
960725_19	omy15	22696254		Summer temperature	0.401	0.24	0.86
613213_52	omy15	25854675		Summer temperature	0.108	0.63	0.94
483230_27	omy15	26179733		Summer temperature	0.050	0.77	1.00
476285_61	omy15	26237921	GPR17_HUMAN;	Summer temperature	0.028	0.84	0.91
266735_60	omy15	33842752		Summer temperature	0.115	0.62	0.80
1075812_26	omy15	35641422	SAX_LITCT;	Summer temperature	0.355	0.28	0.90
1075812_20	omy15	35641422	SAX_LITCT;	Summer temperature	0.355	0.28	0.90
104963_67	omy15	44749437		Summer temperature	0.222	0.44	0.88
1075573_25	omy15	48382098		Summer temperature	0.032	0.83	0.85
933872_49	omy15	49178517		Summer temperature	0.416	0.23	0.92
391551_44	omy16	1101057	ISM1_DANRE;	Summer temperature	0.258	0.39	0.75
1093043_73	omy16	6325228		Summer temperature	0.405	0.24	0.92
925324_37	omy16	15268607		Summer temperature	0.044	0.79	0.88
925324_61	omy16	15268607		Summer temperature	0.125	0.60	0.88
461819_24	omy16	16282391		Summer temperature	0.926	0.00	0.83
400278_51	omy16	19010590		Summer temperature	0.357	0.28	0.95
776381_70	omy16	19051340		Summer temperature	0.357	0.28	0.95
1018813_32	omy16	20191801	DAAF3_DANRE;	Summer temperature	0.357	0.28	0.91
1189573_64	omy16	24653528		Summer temperature	0.115	0.62	0.93
550983_62	omy16	30589751		Summer temperature	0.134	0.58	0.89
1029009_47	omy16	36292057		Summer temperature	0.083	0.69	0.92
922585_64	omy16	37496888		Summer temperature	0.150	0.55	0.90
795318_51	omy16	39787660		Summer temperature	0.691	0.06	0.90
291255_71	omy16	40079617	PKHG5_HUMAN;	Summer temperature	0.097	0.66	1.00
432785_65	omy16	43567699		Summer temperature	0.035	0.82	1.00
747316_44	omy16	44388301		Summer temperature	0.371	0.27	0.95
981106_20	omy16	45647659	CC018_MOUSE;	Summer temperature	0.343	0.30	0.87
292453_60	omy16	48785312	PKHM2_HUMAN;	Summer temperature	0.023	0.86	0.96
945036_20	omy16	48786474	PKHM2_HUMAN;	Summer temperature	0.010	0.92	0.91
421873_34	omy16	54131928		Summer temperature	0.061	0.74	0.95
640890_43	omy16	62095767		Summer temperature	0.339	0.30	0.79
934031_19	omy16	62146178		Summer temperature	0.339	0.30	0.94
1155618_23	omy16	63375642		Summer temperature	0.298	0.34	0.85
788258_64	omy16	64197452		Summer temperature	0.349	0.29	0.75
456238_30	omy16	64250602		Summer temperature	0.454	0.20	0.90
92064_74	omy17	18782026		Summer temperature	0.332	0.31	0.82
942468_60	omy17	18785677		Summer temperature	0.339	0.30	0.82
942468_9	omy17	18785677		Summer temperature	0.339	0.30	0.82
1239779_52	omy17	19605560		Summer temperature	0.033	0.83	0.94
678694_13	omy17	19926752	NAC1_CAVPO;	Summer temperature	0.038	0.81	0.78

Outlier	Chr	Chr_pos	Candidate gene (5 kb)	Environment Variable	p-value	R2	Allele_diff
777293_5	omy17	33436739	BSN_RAT;	Summer temperature	0.045	0.79	0.86
151933_31	omy17	41920671		Summer temperature	0.339	0.30	0.64
733707_11	omy17	42593396		Summer temperature	0.306	0.34	0.88
1124473_9	omy17	64456207	UBA1_RABIT;	Summer temperature	0.128	0.59	0.89
460489_69	omy17	65311406		Summer temperature	0.407	0.24	0.89
877963_61	omy17	70273614		Summer temperature	0.235	0.42	1.00
730749_10	omy18	5835028	TVC_HUMAN;	Summer temperature	0.056	0.76	0.88
684500_10	omy18	13982097		Summer temperature	0.305	0.34	0.88
688708_62	omy18	19032568		Summer temperature	0.022	0.86	1.00
408454_12	omy18	19041375	S100B_HUMAN;	Summer temperature	0.037	0.81	0.75
840777_70	omy18	19067383		Summer temperature	0.339	0.30	0.75
721616_11	omy18	25827563		Summer temperature	0.270	0.38	1.00
222205_61	omy18	29376937		Summer temperature	0.312	0.33	0.94
222205_48	omy18	29376937		Summer temperature	0.312	0.33	0.81
157218_66	omy18	38042510		Summer temperature	0.041	0.80	1.00
1303146_64	omy18	49954334		Summer temperature	0.133	0.58	0.93
945496_48	omy18	58255293		Summer temperature	0.026	0.85	0.92
900900_61	omy19	10472264		Summer temperature	0.357	0.28	0.74
914118_35	omy19	10776741		Summer temperature	0.349	0.29	0.86
548005_65	omy19	18330455		Summer temperature	0.102	0.64	1.00
903110_14	omy19	25631299		Summer temperature	0.642	0.08	0.33
903110_12	omy19	25631299		Summer temperature	0.654	0.08	0.35
495567_13	omy19	33589582		Summer temperature	0.308	0.33	0.90
648147_41	omy19	37732258		Summer temperature	0.137	0.58	1.00
304108_52	omy19	53000175		Summer temperature	0.166	0.52	0.86
304358_28	omy19	53395249		Summer temperature	0.015	0.90	1.00
330442_47	omy20	1359668		Summer temperature	0.190	0.49	0.94
210725_62	omy20	8669592		Summer temperature	0.419	0.23	1.00
844911_45	omy20	26412663		Summer temperature	0.344	0.30	0.95
809732_15	omy20	33568935		Summer temperature	0.055	0.76	1.00
802634_52	omy20	33665034		Summer temperature	0.305	0.34	0.82
802634_53	omy20	33665034		Summer temperature	0.305	0.34	0.82
1307208_56	omy20	34105354		Summer temperature	0.306	0.34	0.94
1437141_65	omy20	34442760		Summer temperature	0.089	0.67	0.93
327989_27	omy21	9030106		Summer temperature	0.219	0.44	0.88
1411478_58	omy21	11889194		Summer temperature	0.354	0.29	1.00
1340057_70	omy21	12270405		Summer temperature	0.370	0.27	0.93
1190996_52	omy21	12909232	FACR1_XENLA;	Summer temperature	0.364	0.28	0.94
111506_37	omy21	15119211		Summer temperature	0.325	0.32	1.00
183630_20	omy21	18374297		Summer temperature	0.209	0.46	0.80
305807_40	omy21	31691004		Summer temperature	0.061	0.74	0.81
343820_60	omy21	32902922		Summer temperature	0.199	0.47	0.94
955637_74	omy21	33060816		Summer temperature	0.343	0.30	0.96
1155837_43	omy21	35557820	UFSP1_MOUSE;	Summer temperature	0.281	0.36	1.00
870524_30	omy21	48865056	PER1_HUMAN;	Summer temperature	0.312	0.33	1.00
870524_46	omy21	48865056	PER1_HUMAN;	Summer temperature	0.312	0.33	0.80

Outlier	Chr	Chr_pos	Candidate gene (5 kb)	Environment Variable	p-value	R2	Allele_diff
387385_45	omy22	8462908		Summer temperature	0.264	0.39	0.87
528036_58	omy22	13248005		Summer temperature	0.258	0.39	0.93
541522_21	omy22	22797506	TFG_HUMAN;	Summer temperature	0.334	0.31	0.88
571587_13	omy22	23632026	SRPX_HUMAN;	Summer temperature	0.142	0.57	1.00
810318_27	omy22	28977007		Summer temperature	0.312	0.33	0.82
284362_34	omy22	28977078		Summer temperature	0.306	0.34	0.94
965813_6	omy22	29311638	5HT1F_RAT;	Summer temperature	0.312	0.33	0.80
547597_12	omy23	1381246		Summer temperature	0.043	0.79	0.83
354845_49	omy23	2829447		Summer temperature	0.041	0.80	0.96
888377_12	omy23	2882309		Summer temperature	0.042	0.80	0.81
1097165_43	omy23	12732810		Summer temperature	0.312	0.33	0.85
1416384_47	omy23	15000252		Summer temperature	0.173	0.51	1.00
161904_7	omy23	15462158		Summer temperature	0.353	0.29	1.00
1308564_46	omy23	32881212		Summer temperature	0.223	0.44	1.00
399409_20	omy23	41039665		Summer temperature	0.230	0.43	1.00
1179293_44	omy23	41084961		Summer temperature	0.415	0.23	1.00
1071884_47	omy23	43093872		Summer temperature	0.281	0.36	0.88
96299_62	omy24	22014095		Summer temperature	0.036	0.81	0.94
548904_14	omy24	34851772	NOS_LYMST;	Summer temperature	0.013	0.90	0.89
263484_20	omy24	37284760		Summer temperature	0.319	0.32	0.94
1229999_23	omy24	39486050		Summer temperature	0.241	0.42	1.00
623386_60	omy25	3391624		Summer temperature	0.097	0.65	0.93
623386_71	omy25	3391624		Summer temperature	0.097	0.65	0.93
208930_27	omy25	13916916		Summer temperature	0.043	0.79	1.00
915533_16	omy25	18879590		Summer temperature	0.232	0.43	1.00
454429_18	omy25	19960092		Summer temperature	0.349	0.29	0.95
267663_74	omy25	23152288		Summer temperature	0.161	0.53	0.88
267663_73	omy25	23152288		Summer temperature	0.163	0.53	0.89
529804_73	omy25	43187198		Summer temperature	0.346	0.29	1.00
1111193_31	omy25	65419329		Summer temperature	0.261	0.39	0.94
1200267_58	omy25	65419477		Summer temperature	0.312	0.33	0.76
1172383_28	omy25	73556718		Summer temperature	0.343	0.30	0.86
1097278_38	omy25	74398833		Summer temperature	0.357	0.28	0.83
1232448_21	omy26	3275231		Summer temperature	0.306	0.34	0.94
1023792_33	omy26	9526680		Summer temperature	0.382	0.26	0.88
958226_5	omy26	9739368	ATS18_MOUSE;	Summer temperature	0.478	0.18	0.94
102758_27	omy26	25328165		Summer temperature	0.316	0.33	0.95
284544_58	omy26	26012843		Summer temperature	0.039	0.81	0.94
687143_17	omy26	26220117		Summer temperature	0.036	0.82	1.00
697447_33	omy26	26252001		Summer temperature	0.048	0.78	0.85
891458_41	omy27	1274364		Summer temperature	0.368	0.27	0.85
286707_50	omy27	8737251		Summer temperature	0.339	0.30	0.83
391656_44	omy27	9922489	TBCEL_RAT;	Summer temperature	0.333	0.31	0.90
746043_42	omy27	11578587		Summer temperature	0.337	0.30	0.82
979686_30	omy27	11580660		Summer temperature	0.312	0.33	0.76
290584_23	omy27	11686018		Summer temperature	0.326	0.31	0.88

Outlier	Chr	Chr_pos	Candidate gene (5 kb)	Environment Variable	p-value	R2	Allele_diff
1169079_7	omy27	13394364	TF21_SCHPO;	Summer temperature	0.800	0.02	0.94
550596_59	omy27	39888848		Summer temperature	0.254	0.40	1.00
962696_13	omy28	5868655	PRP6_BOVIN;	Summer temperature	0.113	0.62	0.86
1278730_17	omy28	10050610		Summer temperature	0.164	0.53	0.93
365942_29	omy28	11593125	ABHD3_HUMAN;	Summer temperature	0.106	0.64	1.00
626736_59	omy28	11627432		Summer temperature	0.091	0.67	0.93
394646_21	omy28	11661062		Summer temperature	0.210	0.46	1.00
1073099_20	omy28	11746944		Summer temperature	0.131	0.59	1.00
823291_35	omy28	11749936		Summer temperature	0.074	0.71	0.95
692810_5	omy28	12027198		Summer temperature	0.204	0.47	0.94
312814_8	omy28	31498567	CPN2_HUMAN;	Summer temperature	0.339	0.30	0.67
241219_5	omy29	36983269		Summer temperature	0.357	0.28	0.79
359430_47	omy29	37675596	F120B_HUMAN;	Summer temperature	0.398	0.24	0.83
537531_13	UNKNOWN	4362		Summer temperature	0.141	0.57	0.95
537531_12	UNKNOWN	4362		Summer temperature	0.144	0.56	0.95
636524_11	UNKNOWN	5293		Summer temperature	0.357	0.28	0.83
826771_53	UNKNOWN	6413		Summer temperature	0.390	0.25	0.90
879243_14	UNKNOWN	9890		Summer temperature	0.185	0.49	1.00
710398_20	UNKNOWN	13254		Summer temperature	0.357	0.28	0.86
554197_59	UNKNOWN	13815		Summer temperature	0.004	0.95	1.00
490925_41	UNKNOWN	14143		Summer temperature	0.357	0.28	0.89
422670_10	UNKNOWN	15260		Summer temperature	0.257	0.39	0.94
808817_74	UNKNOWN	15564		Summer temperature	0.349	0.29	0.78

Note:

Allele_diff is the largest difference in minor allele frequency across all populations.
