# Supplementary material for: Physiological and genomic signatures of evolutionary thermal adaptation in redband trout from extreme climates
Source: Evol Appl. 2018 Jul 20;11(9):1686–99. doi: 10.1111/eva.12672 (PMC6183465; doi:10.1111/eva.12672)
Supplement: Supplementary file 5 [file EVA-11-1686-s005.pdf]

Table S2 Annotation of cardaic traits associated markers

Traits	Ecotype	CHR	Signif. Window_pos	Cadidate genes
T_{AB}	Cold Montane	omy01	23719600-23739400	YTX2_XENLA;
T_{AB}	Cold Montane	omy02	10975000-10993400	
T_{AB}	Cold Montane	omy04	3494400-3505200	
T_{AB}	Cold Montane	omy06	17984200-17994600	PGH1_BOVIN;
T_{AB}	Cold Montane	omy06	19815800-19834800	
T_{AB}	Cold Montane	omy06	42382600-42394200	
T_{AB}	Cold Montane	omy07	62984400-63004200	CAC1D_HUMAN;
T_{AB}	Cold Montane	omy09	42048600-42067800	
T_{AB}	Cold Montane	omy10	62702600-62717800	LRBA_MOUSE;
T_{AB}	Cold Montane	omy11	26293600-26312200	
T_{AB}	Cold Montane	omy12	27551000-27565600	SERPH_CHICK;
T_{AB}	Cold Montane	omy14	9216200-9235000	
T_{AB}	Cold Montane	omy14	11776200-11790800	TM249_HUMAN; TC1A_CAEEL;
T_{AB}	Cold Montane	omy14	58606200-58626000	
T_{AB}	Cold Montane	omy22	28117200-28137000	
$f_{h,max}$@20°C	Cold Montane	omy01	68363600-68377200	
$f_{h,max}$@20°C	Cold Montane	omy06	34363400-34380600	
$f_{h,max}$@20°C	Cold Montane	omy07	62984400-63004200	CAC1D_HUMAN;
$f_{h,max}$@20°C	Cold Montane	omy09	10324800-10344400	SVEP1_HUMAN;
$f_{h,max}$@20°C	Cold Montane	omy09	30563400-30576000	
$f_{h,max}$@20°C	Cold Montane	omy11	10513200-10532000	GP183_RAT;
$f_{h,max}$@20°C	Cold Montane	omy11	56698400-56709000	
$f_{h,max}$@20°C	Cold Montane	omy13	38944600-38954800	
$f_{h,max}$@20°C	Cold Montane	omy13	38945000-38962600	
$f_{h,max}$@20°C	Cold Montane	omy16	12429400-12439600	P4HA1_BOVIN;RTJK_DROME;
$f_{h,max}$@20°C	Cold Montane	omy16	38547200-38562200	
$f_{h,max}$@20°C	Cold Montane	omy16	48954000-48973800	TMM82_XENLA; TC1A_CAEEL;
$f_{h,max}$@20°C	Cold Montane	omy18	30004200-30019400	
$f_{h,max}$@20°C	Cold Montane	omy20	5317000-5327600	
$f_{h,max}$@20°C	Cold Montane	omy21	44514400-44524800	M4K2_HUMAN;
$f_{h,max}$@20°C	Cold Montane	omy22	30353400-30365200	PDCL3_RAT;
peak_$f_{h,max}$	Cold Montane	omy04	70524800-70544600	
peak_$f_{h,max}$	Cold Montane	omy06	13356800-13374600	
peak_$f_{h,max}$	Cold Montane	omy06	13753800-13788600	FBCD1_XENTR;
peak_$f_{h,max}$	Cold Montane	omy06	34363400-34380600	
peak_$f_{h,max}$	Cold Montane	omy07	37773800-37785200	
peak_$f_{h,max}$	Cold Montane	omy08	56030200-56045400	
peak_$f_{h,max}$	Cold Montane	omy09	7577200-7596800	CASQ1_RAT;
peak_$f_{h,max}$	Cold Montane	omy11	10513200-10532000	GP183_RAT;
peak_$f_{h,max}$	Cold Montane	omy11	34042600-34053800	
peak_$f_{h,max}$	Cold Montane	omy18	40305200-40315600	PGCA_RAT;
peak_$f_{h,max}$	Cold Montane	omy19	13286600-13298800	
peak_$f_{h,max}$	Cold Montane	omy21	44178600-44197400	
peak_$f_{h,max}$	Cold Montane	omy24	9939000-9954800	

Traits	Ecotype	CHR	Signif. Window_pos	Cadidate genes
peak_$f_{h,max}$	Cold Montane	omy24	9945000-9958200	
T_{PEAK}	Cold Montane	omy01	40212000-40224000	
T_{PEAK}	Cold Montane	omy02	18445600-18463200	
T_{PEAK}	Cold Montane	omy04	35544600-35554800	
T_{PEAK}	Cold Montane	omy04	35545000-35557600	
T_{PEAK}	Cold Montane	omy04	58363600-58374600	
T_{PEAK}	Cold Montane	omy06	34363400-34380600	
T_{PEAK}	Cold Montane	omy06	70117600-70136800	
T_{PEAK}	Cold Montane	omy07	19773800-19791600	A4_TETFL;
T_{PEAK}	Cold Montane	omy08	18197800-18211400	
T_{PEAK}	Cold Montane	omy09	21543800-21554800	
T_{PEAK}	Cold Montane	omy09	21545000-21558200	
T_{PEAK}	Cold Montane	omy09	27566000-27585800	
T_{PEAK}	Cold Montane	omy10	25901200-25917000	
T_{PEAK}	Cold Montane	omy10	30614400-30625800	RBMX2_MOUSE;GP119_MOUSE;
T_{PEAK}	Cold Montane	omy10	41541600-41554800	NFRKB_MOUSE;
T_{PEAK}	Cold Montane	omy10	41545000-41558600	NFRKB_MOUSE;
T_{PEAK}	Cold Montane	omy11	50753800-50770400	GL1D1_PONAB;
T_{PEAK}	Cold Montane	omy12	33823800-33834800	TRI37_MOUSE;
T_{PEAK}	Cold Montane	omy15	6811200-6823000	
T_{PEAK}	Cold Montane	omy16	21863400-21883200	PUS3_BOVIN;
T_{PEAK}	Cold Montane	omy16	22102400-22118600	
T_{PEAK}	Cold Montane	omy17	19675400-19689000	CARD8_HUMAN;
T_{PEAK}	Cold Montane	omy17	68874600-68894400	
T_{PEAK}	Cold Montane	omy20	32004200-32015200	
T_{PEAK}	Cold Montane	omy21	44178600-44197400	
T_{PEAK}	Cold Montane	omy22	28117200-28137000	
T_{PEAK}	Cold Montane	omy24	14588600-14605400	
T_{PEAK}	Cold Montane	omy29	20268600-20288400	
T_{PEAK}	Cold Montane	omy29	28876000-28894400	
T_{PEAK}	Cold Montane	omy29	31554200-31564200	ADT2_RAT;DFP_MANSE;
T_{AB}	Cool Montane	omy03	20114000-20131800	
T_{AB}	Cool Montane	omy08	19122000-19138400	
T_{AB}	Cool Montane	omy08	23976400-23993800	
T_{AB}	Cool Montane	omy10	42161800-42181400	IF1AX_HUMAN;
T_{AB}	Cool Montane	omy12	40925000-40942000	
T_{AB}	Cool Montane	omy14	49606400-49620600	
T_{AB}	Cool Montane	omy15	2912400-2924800	TRPA1_HUMAN;
T_{AB}	Cool Montane	omy15	4040600-4053400	
T_{AB}	Cool Montane	omy17	65664200-65674400	
T_{AB}	Cool Montane	omy19	25760000-25777000	
T_{AB}	Cool Montane	omy21	50571400-50585000	
T_{AB}	Cool Montane	omy25	24222800-24235400	
$f_{h,max}$@20°C	Cool Montane	omy01	4601400-4621200	WDR47_HUMAN;
$f_{h,max}$@20°C	Cool Montane	omy01	26714600-26726200	
$f_{h,max}$@20°C	Cool Montane	omy02	32936000-32954800	OSBL5_HUMAN;

Traits	Ecotype	CHR	Signif. Window_pos	Cadidate genes
$f_{h,max}$@20°C	Cool Montane	omy02	32945000-32955600	
$f_{h,max}$@20°C	Cool Montane	omy03	18374200-18384200	
$f_{h,max}$@20°C	Cool Montane	omy04	22675200-22688800	
$f_{h,max}$@20°C	Cool Montane	omy06	24245400-24256600	
$f_{h,max}$@20°C	Cool Montane	omy10	13892000-13906200	
$f_{h,max}$@20°C	Cool Montane	omy12	2301600-2316200	
$f_{h,max}$@20°C	Cool Montane	omy12	39786800-39799400	
$f_{h,max}$@20°C	Cool Montane	omy12	58110200-58125400	
$f_{h,max}$@20°C	Cool Montane	omy13	26653800-26672800	
$f_{h,max}$@20°C	Cool Montane	omy13	29681400-29693800	
$f_{h,max}$@20°C	Cool Montane	omy15	14839200-14852200	
$f_{h,max}$@20°C	Cool Montane	omy15	32756600-32768200	
$f_{h,max}$@20°C	Cool Montane	omy16	43400800-43420600	SYPH_HUMAN;CAC1F_HUMAN;
$f_{h,max}$@20°C	Cool Montane	omy16	44752800-44762800	F19A1_HUMAN;
$f_{h,max}$@20°C	Cool Montane	omy16	46158000-46169000	
$f_{h,max}$@20°C	Cool Montane	omy18	5185800-5198000	
$f_{h,max}$@20°C	Cool Montane	omy19	23233000-23245000	
$f_{h,max}$@20°C	Cool Montane	omy21	50571400-50585000	
$f_{h,max}$@20°C	Cool Montane	omy22	15239400-15251400	
$f_{h,max}$@20°C	Cool Montane	omy22	39488600-39502400	
$f_{h,max}$@20°C	Cool Montane	omy22	44336200-44354800	
$f_{h,max}$@20°C	Cool Montane	omy22	44345000-44356000	
$f_{h,max}$@20°C	Cool Montane	omy23	16204400-16215800	HMX2_MOUSE;
$f_{h,max}$@20°C	Cool Montane	omy23	43878600-43898400	
$f_{h,max}$@20°C	Cool Montane	omy23	44066600-44078800	BIG3_HUMAN;
$f_{h,max}$@20°C	Cool Montane	omy24	9450600-9462600	ZFYV1_MOUSE;
peak_$f_{h,max}$	Cool Montane	omy01	11770600-11789000	TRM13_MOUSE;ODB2_BOVIN;
peak_$f_{h,max}$	Cool Montane	omy01	45524200-45534600	
peak_$f_{h,max}$	Cool Montane	omy01	62321600-62336200	
peak_$f_{h,max}$	Cool Montane	omy04	6041600-6051800	
peak_$f_{h,max}$	Cool Montane	omy04	22675200-22688800	
peak_$f_{h,max}$	Cool Montane	omy07	11000000-11010600	
peak_$f_{h,max}$	Cool Montane	omy08	45155400-45166600	
peak_$f_{h,max}$	Cool Montane	omy08	57925400-57939400	
peak_$f_{h,max}$	Cool Montane	omy09	46069600-46079600	GBB1_DANRE;
peak_$f_{h,max}$	Cool Montane	omy15	50539000-50550400	
peak_$f_{h,max}$	Cool Montane	omy16	5361600-5381400	
peak_$f_{h,max}$	Cool Montane	omy16	23909200-23926000	
peak_$f_{h,max}$	Cool Montane	omy16	32598600-32610400	
peak_$f_{h,max}$	Cool Montane	omy20	7256600-7276400	ITB4_RAT;ITB4_MOUSE;
peak_$f_{h,max}$	Cool Montane	omy22	2597400-2613800	
peak_$f_{h,max}$	Cool Montane	omy22	2826600-2839200	
peak_$f_{h,max}$	Cool Montane	omy22	3436200-3450200	
peak_$f_{h,max}$	Cool Montane	omy22	45478000-45497400	ERCC1_BOVIN;
peak_$f_{h,max}$	Cool Montane	omy23	37971400-37990000	PGRC1_PONAB;
peak_$f_{h,max}$	Cool Montane	omy25	62189400-62201000	

Traits	Ecotype	CHR	Signif. Window_pos	Cadidate genes
peak_$f_{h,max}$	Cool Montane	omy28	2303400-2320400	RHG12_HUMAN;
peak_$f_{h,max}$	Cool Montane	omy28	20688000-20707800	
peak_$f_{h,max}$	Cool Montane	omy29	18374200-18388400	
T_{PEAK}	Cool Montane	omy01	37344800-37354800	
T_{PEAK}	Cool Montane	omy01	37345000-37357800	
T_{PEAK}	Cool Montane	omy01	70006800-70017000	
T_{PEAK}	Cool Montane	omy03	20981200-21001000	
T_{PEAK}	Cool Montane	omy03	65743600-65753600	
T_{PEAK}	Cool Montane	omy04	2183000-2194000	
T_{PEAK}	Cool Montane	omy04	10777600-10804200	
T_{PEAK}	Cool Montane	omy04	13071000-13083200	
T_{PEAK}	Cool Montane	omy05	5806200-5826000	
T_{PEAK}	Cool Montane	omy05	55892600-55904200	
T_{PEAK}	Cool Montane	omy06	59684000-59701400	
T_{PEAK}	Cool Montane	omy07	34415000-34427200	GBB1_DANRE;
T_{PEAK}	Cool Montane	omy08	41752200-41765800	
T_{PEAK}	Cool Montane	omy10	37220000-37239600	
T_{PEAK}	Cool Montane	omy11	56988800-57006000	
T_{PEAK}	Cool Montane	omy12	43585000-43599200	
T_{PEAK}	Cool Montane	omy12	81472600-81484400	
T_{PEAK}	Cool Montane	omy15	5185600-5196200	
T_{PEAK}	Cool Montane	omy15	7670200-7690000	PCY1B_MOUSE;
T_{PEAK}	Cool Montane	omy15	30418200-30435400	
T_{PEAK}	Cool Montane	omy15	57636800-57648800	
T_{PEAK}	Cool Montane	omy16	15289000-15300400	
T_{PEAK}	Cool Montane	omy16	44477000-44496400	
T_{PEAK}	Cool Montane	omy17	46201800-46214800	S61A2_ONCMY;
T_{PEAK}	Cool Montane	omy17	59447000-59459600	
T_{PEAK}	Cool Montane	omy19	10489400-10502000	CEP44_XENLA;
T_{PEAK}	Cool Montane	omy19	41580000-41590000	PUA1C_SALSA;
T_{PEAK}	Cool Montane	omy19	50535800-50552000	
T_{PEAK}	Cool Montane	omy20	10009600-10028200	
T_{PEAK}	Cool Montane	omy20	40046000-40058200	
T_{PEAK}	Cool Montane	omy21	2536400-2547200	
T_{PEAK}	Cool Montane	omy21	24227200-24242200	SO1C1_HUMAN;
T_{PEAK}	Cool Montane	omy22	3895600-3907600	
T_{PEAK}	Cool Montane	omy22	11199800-11216600	
T_{PEAK}	Cool Montane	omy23	11956000-11966000	
T_{PEAK}	Cool Montane	omy23	26593400-26607600	
T_{PEAK}	Cool Montane	omy23	40074600-40089600	
T_{PEAK}	Cool Montane	omy23	47722400-47735200	
T_{PEAK}	Cool Montane	omy24	18185000-18204600	
T_{PEAK}	Cool Montane	omy25	19279800-19317000	ADA1B_MESAU;
T_{PEAK}	Cool Montane	omy25	31353000-31372800	SCND3_HUMAN;
T_{PEAK}	Cool Montane	omy25	53791200-53804000	
T_{PEAK}	Cool Montane	omy29	24073400-24084600	

Traits	Ecotype	CHR	Signif. Window_pos	Cadidate genes
T_{PEAK}	Cool Montane	omy29	25760000-25774800	
T_{AB}	Desert	omy05	69099800-69115200	
T_{AB}	Desert	omy10	15851600-15862800	
T_{AB}	Desert	omy14	64373000-64384000	
T_{AB}	Desert	omy15	11532400-11542600	ABHDA_HUMAN;
T_{AB}	Desert	omy15	18676000-18689200	
T_{AB}	Desert	omy15	26757400-26768000	DLGP1_HUMAN;
T_{AB}	Desert	omy18	35427200-35443200	
T_{AB}	Desert	omy29	20268600-20288400	
$f_{h,max}$@20°C	Desert	omy18	36616000-36628800	
$f_{h,max}$@20°C	Desert	omy20	28854600-28874200	
peak_$f_{h,max}$	Desert	omy06	35391200-35403400	
peak_$f_{h,max}$	Desert	omy25	28002600-28018600	RAN_SALSA;
T_{PEAK}	Desert	omy01	43374400-43384600	
T_{PEAK}	Desert	omy01	57319000-57337200	KCIP2_MUSPF;
T_{PEAK}	Desert	omy02	71593000-71604800	REP15_HUMAN;
T_{PEAK}	Desert	omy02	71909200-71924000	
T_{PEAK}	Desert	omy03	16842000-16853000	
T_{PEAK}	Desert	omy04	16956200-16970800	ENL_HUMAN;
T_{PEAK}	Desert	omy06	7113000-7126000	
T_{PEAK}	Desert	omy06	42911600-42921600	
T_{PEAK}	Desert	omy08	71277600-71297400	HMHA1_HUMAN;
T_{PEAK}	Desert	omy13	11876800-11894800	TITIN_MOUSE;
T_{PEAK}	Desert	omy13	22089600-22106800	
T_{PEAK}	Desert	omy18	35897000-35910000	C3AR_DANRE;TCB1_CAEBR;

Note:
T_{AB} : Arrhenius breakpoint temperature
Tpeak : Temperature of peak_fhmax
$f_{h,max}$@20°C: maximun heart rate at 20°C
peak_$f_{h,max}$: peak maximun heart rate
