# Supplementary material for: Physiological and genomic signatures of evolutionary thermal adaptation in redband trout from extreme climates
Source: Evol Appl. 2018 Jul 20;11(9):1686–99. doi: 10.1111/eva.12672 (PMC6183465; doi:10.1111/eva.12672)
Supplement: Supplementary file 6 [file EVA-11-1686-s006.docx]

Table S3. candidate genes of redband trout thermal adaptation from previous studies.

| Publication | Method | Candidate gene |
| --- | --- | --- |
| Narum et al., 2010 | 96 SNP chip | GLDH (Glutamate dehydrogenase) |
|  | G × E associations | STAT (Signal transducers and activator of transcription) |
|  |  | ALDOB (Aldolase B Toll-like) |
|  |  | TLR5 (Toll-like receptor 5) |
|  |  |  |
| Narum et al., 2013 | 10000 SNPs | HSP47 |
|  | (RAD) | Immune response MCH genes |
|  | GWAS | HSF2 |
|  |  | HSC71 |
|  |  | Na–K-ATPase-a3 |
|  |  |  |
| Chen et al., 2018 | 5903 SNPs (RAD) | LDH-B (lactate dehydrogenase b) |
|  | F_ST_ Outlier loci | CAMKK2 (calcium calmodulin-dependent protein kinase type 1d) |
|  |  | HSP40 (dnaj b6) |
|  |  | GOT1B (golgi transport 1b) and |
|  |  | CFC1 (cripto, FRL-1, cryptic family 1) |
